# Correction: Assessment of staffing needs for physicians and nurses at Upazila health complexes in Bangladesh using WHO workload indicators of staffing need (WISN) method

**DOI:** 10.1136/bmjopen-2019-035183corr1

**Published:** 2020-03-16

Joarder T, Tune SNBK, Nuruzzaman M, *et al*. Assessment of staffing needs for physicians and nurses at Upazila health complexes in Bangladesh using WHO workload indicators of staffing need (WISN) method. *BMJ Open* 2020;10:e035183. doi: 10.1136/bmjopen-2019-035183

This article was previously published with an error.

Reference 29 is corrected as: Park CS-Y. Optimizing staffing, quality, and cost in home healthcare nursing: theory synthesis. *J Adv Nurs* 2017;73:1838–47.

