# Supplementary material for: Assessment of staffing needs for physicians and nurses at Upazila health complexes in Bangladesh using WHO workload indicators of staffing need (WISN) method
Source: BMJ Open. 2020 Feb 13;10(2):e035183. doi: 10.1136/bmjopen-2019-035183 (PMC7045064; doi:10.1136/bmjopen-2019-035183)
Supplement: Author's manuscript [file bmjopen-2019-035183.draft_revisions.pdf]

BMJ Open is committed to open peer review. As part of this commitment we make the peer review history of every article we publish publicly available.

When an article is published we post the peer reviewers' comments and the authors' responses online. We also post the versions of the paper that were used during peer review. These are the versions that the peer review comments apply to.

The versions of the paper that follow are the versions that were submitted during the peer review process. They are not the versions of record or the final published versions. They should not be cited or distributed as the published version of this manuscript.

BMJ Open is an open access journal and the full, final, typeset and author-corrected version of record of the manuscript is available on our site with no access controls, subscription charges or pay-per-view fees (http://bmjopen.bmj.com).

If you have any questions on BMJ Open's open peer review process please email info.bmjopen@bmj.com

BMJ Open

An assessment of staffing needs for physicians and nurses at Upazila Health Complexes in Bangladesh using WHO workload indicators of staffing need (WISN) method

Journal:	*BMJ Open*
Manuscript ID	bmjopen-2019-035183
Article Type:	Original research
Date Submitted by the Author:	21-Oct-2019
Complete List of Authors:	Joarder, Taufique; BRAC University, BRAC James P Grant School of Public Health; FHI 360, Bangladesh Office<br>Tune, Samiun; BRAC James P Grant School of Public Health, BRAC University, Public Health;<br>Nuruzzaman, Mahmoud; World Health Organization, Country Office, Dhaka, Bangladesh<br>Alam, Sabina; Government of Bangladesh Ministry of Health and Family Welfare, Health Services Division<br>Cruz, Valeria; World Health Organization, Country Office for Bangladesh<br>Zapata, Tomas; SEARO
Keywords:	Human resource management < HEALTH SERVICES ADMINISTRATION & MANAGEMENT, Health policy < HEALTH SERVICES ADMINISTRATION & MANAGEMENT, PUBLIC HEALTH, HEALTH SERVICES ADMINISTRATION & MANAGEMENT

SCHOLARONE™
Manuscripts

I, the Submitting Author has the right to grant and does grant on behalf of all authors of the Work (as defined in the below author licence), an exclusive licence and/or a non-exclusive licence for contributions from authors who are: i) UK Crown employees; ii) where BMJ has agreed a CC-BY licence shall apply, and/or iii) in accordance with the terms applicable for US Federal Government officers or employees acting as part of their official duties; on a worldwide, perpetual, irrevocable, royalty-free basis to BMJ Publishing Group Ltd ("BMJ") its licensees and where the relevant Journal is co-owned by BMJ to the co-owners of the Journal, to publish the Work in this journal and any other BMJ products and to exploit all rights, as set out in our licence.

The Submitting Author accepts and understands that any supply made under these terms is made by BMJ to the Submitting Author unless you are acting as an employee on behalf of your employer or a postgraduate student of an affiliated institution which is paying any applicable article publishing charge ("APC") for Open Access articles. Where the Submitting Author wishes to make the Work available on an Open Access basis (and intends to pay the relevant APC), the terms of reuse of such Open Access shall be governed by a Creative Commons licence – details of these licences and which Creative Commons licence will apply to this Work are set out in our licence referred to above.

Other than as permitted in any relevant BMJ Author's Self Archiving Policies, I confirm this Work has not been accepted for publication elsewhere, is not being considered for publication elsewhere and does not duplicate material already published. I confirm all authors consent to publication of this Work and authorise the granting of this licence.

For peer review only

**Title page

An assessment of staffing needs for physicians and nurses at Upazila Health Complexes in Bangladesh using WHO workload indicators of staffing need (WISN) method

Taufique Joarder[1, 2], Samiun Nazrin Bente Kamal Tune[1*], Md Nuruzzaman[3], Sabina Alam[4], Valeria de Oliveira Cruz[5], Tomas Zapata[5]

[1]BRAC James P Grant School of Public Health, BRAC University, Dhaka

[2]FHI360, Dhaka, Bangladesh

[3]World Health Organization Country Office for Bangladesh, Dhaka

[4]Health Services Division, Ministry of Health and Family Welfare, Government of Bangladesh

[5]World Health Organization South-East Asia Regional Office, New Delhi

Email addresses:

TJ: taufiquejoarder@gmail.com

SNBKT: samiun.tune@gmail.com

MN: nuruzzamanm@who.int

SA: alam6350@gmail.com

VOC: deoliveiracruzv@who.int

TZ: zapatat@who.int

[*]Corresponding author

For peer review only

Abstract

Objective This study aimed to assess the current workload and staffing need of Physicians and Nurses for delivering optimum health care services at the Upazila Health Complexes (UpHCs) in Bangladesh.

Design Mixed-methods, combining qualitative (e.g., document reviews, key informant interviews, in-depth interviews, observations) and quantitative methods (time-motion survey)

Setting Study was conducted in 24 health facilities of Bangladesh. However, UpHCs being the nucleus of primary health care in Bangladesh, this manuscript limits itself to reporting the findings from the providers at four UpHCs under this project.

Participants 18 Physicians and 51 Nurses, males and females.

Primary outcome measures Workload components were defined based on inputs from five experts, refined by nine service providers. Using WHO WISN software, standard workload, category allowance factor, individual allowance factor, total required number of staff, WISN Difference, and WISN Ratio were calculated.

Results: Physicians have Very High (WISN Ratio 0.43) and Nurse High (WISN Ratio 0.69) workload pressure. 50% of Nurses' time are occupied with support activities, instead of nursing care. There are different workloads among the same staff category in different health facilities. If only the vacant posts are filled, the workload is reduced. In fact, sanctioned number of Physicians and Nurses is more than actual need.

Conclusions: It is evident that high workload pressures prevail for Physicians and Nurses at the UpHCs. This reveals high demand for these health workforces in the respective sub-districts. WISN method can aid the policy-makers in optimizing utilization of existing human resources. Therefore, the government should adopt flexible health workforce planning and recruitment policy

For peer review only - http://bmjopen.bmj.com/site/about/guidelines.xhtml

to manage the patient load and disease burden. WISN should thus be incorporated as a planning tool for health managers. There should be a regular review of health workforce management decisions, and these should be amended based on periodic reviews.

Strengths and limitations

- Time-motion study helped the research team gain a better understanding of the service context of the staffs. When the key informants or experts suggested an unrealistic activity standard, we presented them the time-motion findings and helped them suggest more context sensitive standards.
- Using both qualitative and quantitative methods for primary data collection complemented each other for bringing data accuracy.
- Technical inputs from the WHO technical experts in WISN application in other countries, and officials of the Ministry of Health improved the data quality as they were directly involved in quality checks at the field level.
- One limitation was that some service statistics data, essential for establishing standard workloads, were unavailable.
- Due to lack of scope in WISN methodology, patient engagement was minimal.

Keywords

Human Resources for Health, Workforce Management, Workload Indicators of Staffing Need, Bangladesh, Upazila Health Complex, Health Systems

For peer review only - http://bmjopen.bmj.com/site/about/guidelines.xhtml

Background

Shortage of Human Resources for Heath (HRH) has been one of the major challenges faced by the health system and globally, more than 90 countries are haunted by this crisis. According to International Labor Organization (ILO), there are on average 34.5 health workers per 10,000 population and about one third of the world's population lack access to health care because of shortage of health workforce[1]. According to the Global strategy on human resources for health: workforce 2030, the estimated global shortage of skilled health workers will be around 18 million by 2030[2]. This problem has reached a critical stage in three (Bangladesh, Myanmar and Bhutan) South-East Asia Region countries with <23 health workers (doctors, nurses and midwives) per 10,000 population, limiting access to health services[3].

Improving health workers' performance and productivity is vital for better health service provision in the country. Poor performance of the health workers has been reported in the literature resulting from too few staff, or staff not providing care according to standards[4, 5, 6]. The extent of the shortage is reflected in health worker density rates and workforce vacancy rates, and its impact in health system performance indicators. Factors that contribute to poor performance of health workers include limited employment opportunities and low salaries; poor working conditions, weak support and supervision, and limited opportunities for professional development[7].

Bangladesh's health workforce scenario is characterized by "shortage, inappropriate skill mix and inequitable distribution"[8, 9]. Equitable access to skilled and motivated health worker in a functional health system is essential for achieving Universal Health Coverage (UHC) and the Sustainable Development Goals[10]. In 2015, Government of Bangladesh (GoB) approved the Bangladesh

For peer review only - http://bmjopen.bmj.com/site/about/guidelines.xhtml

Health Workforce Strategy which affirms government's vision of equitable availability of skilled, motivated and responsive health workforce in adequate numbers across the country[11]. However, there is lack of comprehensive, nationally representative data on HRH workload and optimum staff need in health care facilities in Bangladesh. A small scale qualitative study found overwhelming workload as one of the critical components that hinders retention of doctors and nurses at rural healthcare facilities in Bangladesh[12]. Another policy analysis on retention of HRH (physicians and nurses) also found that deficiency of adequate workforce and consequent high workload acted as a deterrent against rural retention[13].

Workload management is very important for any country or institution to deliver quality services, retain staffs and reduce turnover[14]. Even the seminal document on HRH, "Global strategy on human resources for health: Workforce 2030", emphasized on developing country level workforce strategies, drawing on workload analysis studies[15]. Such studies can provide detailed insight into the current state of workload in a system, coping strategies of the staff for regular extra work pressure, causes behind the excessive workloads, and ways to deal with it. This study aimed to fill-in this knowledge gap with respect to workload and optimum staff need for physicians and nurses at the Upazila or Sub-district level (i.e., at Upazila Health Complex [UpHC]). It is expected that this workload analysis will contribute in improving performance, ensuring quality of services, and facilitating uninterrupted service delivery through efficient management of staff.

Workload Indicator of Staffing Need (WISN) overview

WHO developed the Workload Indicator of Staffing Need (WISN) method in 1998, which was later updated based on learning from implementation in different countries. This method is simple,

For peer review only - http://bmjopen.bmj.com/site/about/guidelines.xhtml

useful and time-saving, which was borrowed from the industrial sector for use in the health sector by Peter Shipp in 1984. The result is expressed in terms of differences and ratios, the former indicating worker shortage or surplus, and the latter workload pressure experienced by the staff [16].

WISN results help in human resource decision-making in several ways (Figure 1). For example, recruitment and transfer of HRH can be based on geographic comparison of WISN ratios, staffing of health facilities can be informed by WISN-based workload projection, etc.

Methods

Study design

We followed the updated WISN manual[16], but contextualized it for Bangladeshi setting. The WISN steps have been summarized in Figure 2. The research project was developed based on close collaboration among and mutual insights from three types of committees:

1. **Steering Committee (SC):** The SC was consisting 13 members, established by the Ministry of Health and Family Welfare (MOHFW) with membership from senior government officials (seven): WHO official (one); professional organization of the physicians, Bangladesh Medical Association (one); and relevant academia (four) such as BRAC University (two persons), Bangladesh University of Health Sciences, and Center for Medical Education. All seven senior government officials were directly involved in decision making regarding daily management of the health workforce in their respective departments. WHO officer was there to respond to WISN related technical issues and application. All four academicians were part of the committee because they had expertise in their respective areas (i.e. education, policy making, curriculum development and performance assessment) of the health workforce. They were also

For peer review only - http://bmjopen.bmj.com/site/about/guidelines.xhtml

well known in the community of scientific writing and academic teaching. The role of the SC was to guide and endorse the overall study based on the WISN strategy and its implementation.

2. **Technical Taskforce (TT):** TT was responsible for guiding the implementation of the WISN process. Researchers from implementing research institution (the school of public health of a Bangladeshi university, BRAC University); and experts from WHO Bangladesh Country Office; an international non-governmental organization (NGO), Save the Children; and another university, Bangladesh University of Health Sciences served in the Taskforce.
3. **Expert Working Groups (EWG):** There were multiple EWGs, one for each of the following professional groups: General Physicians (Medical Officer [MO], Emergency Medical Officer [EMO], Residential Medical Officer [RMO]), and Nursing Staff (Senior Staff Nurse, Nursing Supervisor). The respective EWG defined the workload components and set activity standard for the specific staff category.

The qualitative part of this research involved document reviews, key informant interviews (KII) with policy level persons related to HRH issues in Bangladesh (mostly from among SC and EWG members), in-depth interviews (IDI) with individual service providers (e.g., physicians, nurses, etc. working in UpHCs under this study), and observations. The quantitative component involved time-motion survey which is a work measurement technique for recording the times and rates of working for the elements of a specific job though observing a subject continuously or in a certain period of time[17]. Time motion data served as a guide to determine the activity standard for WISN analyses.

For peer review only - http://bmjopen.bmj.com/site/about/guidelines.xhtml

Study duration, setting and population

The research continued from July to November 2017 and was carried out in two pre-selected Districts of Bangladesh (Figure 3):

- Jhenaidah, located in south-western part of Bangladesh, under Khulna Division
- Moulvibazar, located in north-eastern part of Bangladesh, under Sylhet Division

The selection of the districts was made by the Human Resources Unit, Ministry of Health and Family Welfare **in collaboration with the development partners (Save the Children and WHO Bangladesh) in a workshop nationally organized in November 2016. Later the selection of the UpHCs was made in consultation with the SC, taking into consideration some performance indicators such as** number of beds, number of total deliveries, number of live births, bed occupancy rates as well as patient load including the number of outpatient visits. **From each district, two highest performing UpHCs were included in the study**[18]. Thus, Shailkupa and Kotchandpur UpHCs were selected from Jhenaidah District and Kulaura and Sreemangal from the Moulvibazar. Because future applications need to be based on optimum quality standards, the highest performing UpHCs were selected to serve as a model for other health facilities. From each of these four UpHCs, physicians (Medical Officers and Residential Medical Officers) and nurses (Senior Staff Nurse and Nursing Supervisor) were included for workload analysis. A total of 24 health facilities from the two districts were studied. This included two district hospitals, four UpHCs, two Maternal and Child Welfare Centers, eight Union Sub Centers, eight Union Health and Family Welfare Centers, and eight Community Clinics. This manuscript reported findings from all the four UpHCs under this study, as UpHCs are the nucleus of the primary health care delivery in Bangladesh, serving the rural population.

For peer review only - http://bmjopen.bmj.com/site/about/guidelines.xhtml

Sampling strategy

Qualitative part

Documents for review were selected based on the suggestions from the experts (members of SC, TT, and EWG), supplemented by reference tracking of government reports and published literature on HRH of Bangladesh. Key Informants were selected on the principles of purposive sampling[19], supplemented by snowball sampling (i.e., based on the reference or suggestion from the key informants). IDI respondents were selected through purposive sampling, based on the respondent's seniority and designation (e.g., Residential Medical Officer, Nursing Supervisors, etc.). These respondents were practicing individuals and had more than 10 years of experience and played a supervisory role in their respective health facilities.

Quantitative part

For time-motion study, time sampling was done for each consenting staff available during the data collection period. Field Data Collectors (FDCs) observed each staff twice for 45-minutes duration, once during the first half of their service duration and again during the second half. This was done to minimize the bias in the time data due to the patient load (assuming higher patient load in the first half and lower in the second).

For peer review only - http://bmjopen.bmj.com/site/about/guidelines.xhtml

Tool development, pretest, training of data collectors, agreement test

For qualitative data collection, semi-structured guidelines, including that for document reviews, KIIs, IDIs, and observation, were developed. For the time-motion study, structured observation tool was designed. The structured observation tools contained three sections:

1. Background information of observation setting and the person under observation
2. Time-motion data sheet (containing three columns: type of activity, time spent in minutes, and remarks)
3. Example of the activities (health service activities, support activities, and additional activities)

Health service activities, according to WISN manual, are performed by all members of the staff category and regular service statistics are available for them for example, obstetrical service, emergency service, outpatient service, etc. Support activities are also performed by all members of the staff category, but regular service statistics are not available for them for example, record keeping and reporting, attending meetings, instrument sterilization, etc. Additional activities are performed by only certain members of the staff category (e.g., the supervisor or a senior member), and regular service statistics are not available for them for example, duty roster preparation, preparing staff evaluation reports, supervision of cleanliness, etc.[17].

The examples of health service activities were primarily drawn from the list of the activities mentioned in the Essential Services Package (ESP)[20] for respective health facility type. Since service statistics were not available according the ESP activity list, it was adjusted for the local context with inputs from the respondents (through KII, followed by IDI), in alignment with the

For peer review only - http://bmjopen.bmj.com/site/about/guidelines.xhtml

availability of service statistics. An ‘hourglass’ approach was adopted for defining the workload components based on the ESP (Figure 4). Tools were pre-tested in a UpHC near Dhaka, before applying for actual data collection. Qualitative tools were also pre-tested through mock IDIs and KIIs. The pre-testing exercise was followed by the training of the Field Supervisors (FSs) and Field Data Collectors (FDCs).

Data collection and quality control

At first, FSs were sent to respective districts to orient the personnel on the project, seek support, and assess the availability of the service statistics. The FSs spent one week in each district and conducted qualitative observation of the service provision at the UpHCs, to gain a firsthand understanding of the context.

In the second step, we conducted five KIIs to define workload components, in light of the ESP document’s standard of services by facility level[20]. The workload components were further refined based on IDIs with a total of eight physicians and eight nurses.

In the third step, the FDCs, under the supervision of FSs, conducted time-motion study, using a mobile device (SurveyCTO software). During the time FDCs were collecting time-motion data, the FS, in addition to supervising the FDCs, conducted additional IDIs and collected data on available working time; time required for health service, support, and additional activities; and service statistics.

For peer review only - http://bmjopen.bmj.com/site/about/guidelines.xhtml

The next step was data validation and set activity standards. Primary data validation was done through phone calls made to the services providers, and health facility statisticians. Secondly, these were shared with the SC and TT members. Finally, interviews were conducted with the EWG members to finalize the activity standards (Table 1).

Table 1 Number of interviewees representing EWG of staff categories

EWG representing staff category	Number
Physicians: MO, EMO, RMO	2
Nurses: Nursing Supervisor, Senior Staff Nurse	7
Total	9

We presented the time-motion findings to the EWG members and requested them to be as realistic as possible in suggesting activity standards. We also requested them to account for the variability of patient load due to factors such as seasonality, timing of day, facility catchment population, etc. Finally, through multiple meetings, debates and deliberations, the activity standard was finalized, taking into account the information from the IDIs and KIIs, and inputs from the EWG members (Table 2).

Table 2 Service standard for Physicians and Nurses in UpHCs, Bangladesh, 2017

Activities	Service standard		Unit
	Physician	Nurse	

For peer review only - http://bmjopen.bmj.com/site/about/guidelines.xhtml

Obstetrical Service (Caesarean Section)[a]	90	90	min/patient
Obstetrical Service (Normal Delivery)	60	120	min/patient
Newborn Management	15	15	min/inpatient day
Emergency Service	15	Not applicable	min/patient
IMCI/ Nutritional Service	15	15	min/patient
OPD Service (including NCD management)	10	Not applicable	min/patient
First ANC	20	20	min/patient
Follow-up ANC	10	10	min/patient
PNC	15	15	min/patient
Indoor Services (Round, including minor bedside procedures)[b]	25.65	34	min/inpatient day
Bedside patient care	Not applicable	17	min/inpatient day
Patient admission and discharge	Not applicable	20	min/inpatient day
Death certification and associated arrangements	20	30	min/patient

258 **Note:** [a]For Nurses: Assist Obstetrical Service (Caesarean Section); [b]For Nurses: Indoor Services
259 (Round with Physician)
260 # IMCI: Integrated Management of Childhood Illness; OPD: Out Patient Department; NCD: Non-
261 communicable Diseases; ANC: Antenatal Care; PNC: Postnatal Care.

For peer review only

In order to ensure the quality of data, the Principal Expert (lead author of this article), Co-experts (two co-experts – one was leading data collection and the other was leading data quality check and reporting), WHO team consisting of national and international technical experts, and officials from the Human Resource Branch of Ministry of Health and Family Welfare (MoHFW) conducted field visits to each study District and the health facilities therein. During the time-motion data collection period, the Co-experts monitored the data and their geographic location in real-time. They also regularly checked the consistency of the data. Our field based data collection team saved contact information of all the respondents; so, in case of any confusion or need for clarification, the Co-experts called the respondents over phone.

Data management and analysis

The first analytic step was to estimate available working time of the staffs. This is the time a health worker has available in one year to do his or her work, taking into account authorized and unauthorized absences[17]. For all categories of staff, a uniform number of weeks per year (52 weeks), working days in one week (six days), possible working days in one year (52 * 6 = 312 days) were estimated. Next, absent days, such as public holidays (20 days), earned leave (average for each staff category, based on Health Management Information System data), and casual leave (20 days) were deducted to obtain the annual working time in days. Multiplying this with daily working hours (six hours per day), we obtained annual working time in hours.

For peer review only - http://bmjopen.bmj.com/site/about/guidelines.xhtml

Workload components were defined through the inputs from the key informants; activity standards were also set through the interviews with the EWG members. An activity standard is the time necessary for a well-trained, skilled and motivated worker to perform an activity to professional standards in the local circumstances[17]. Both service standards (for health service activities), category allowance standards (for support activities), and individual allowance standards (for additional activities) were determined in the same way.

The next analytic step was to establish standard workload, which was done by dividing the annual working time by unit time of health service activities. A standard workload is the amount of work within a health services workload component that one health service provider can do in a year hypothetically[17]. Then category allowance factor and individual allowance factors were calculated using the following formula, respectively:

Category allowance factor = 1 / {1 – (Total category allowance standard / 100)}

Individual allowance factor = Total individual allowance standard / Available working time in hours

Next, exact number of required staff was calculated by the following formula:

Total required number of staff = (Staff needed for health service activity * Category allowance factor) + Individual allowance factor

The fractional results were rounded up or down, following the guideline provided in the WISN manual[17]:

- 1.0 – 1.1 is rounded down to 1 and >1.1 – 1.9 is rounded up to 2
- 2.0 – 2.2 is rounded down to 2 and >2.2 – 2.9 is rounded up to 3
- 3.0 – 3.3 is rounded down to 3 and >3.3 – 3.9 is rounded up to 4

For peer review only - http://bmjopen.bmj.com/site/about/guidelines.xhtml

- 4.0 – 4.4 is rounded down to 4 and >4.4 – 4.9 is rounded up to 5
- 5.0 – 5.5 is rounded down to 5 and >5.5 – 5.9 is rounded up to 6

Finally, based on the existing number of staff in the respective health facilities, we calculated both the difference (current number of staff – required number of staff by WISN), and the ratio (current number of staff / required number of staff by WISN). The WISN difference indicates whether the health facilities are relatively understaffed (i.e., when the WISN difference is negative), overstaffed (i.e., when the WISN difference is positive), or balanced (i.e., when the WISN difference is zero). The WISN Ratio indicates whether the staffs are experiencing high workload (i.e., when the WISN Ratio is lower than one), low workload (i.e., when the WISN Ratio is higher than one), or normal workload (i.e., when the WISN Ratio is equal to one). For this calculation, we used the de-facto number of current staff, i.e., the number of staff that we actually found working in the health facilities during our data collection period; not the number shown in the office records or statistics.

Ethical considerations

Ethical approval for this study was obtained from the Ethical Review Committee (ERC) of BRAC James P Grant School of Public Health, BRAC University. All ethical principles, were strictly adhered to. Appropriate consent process was followed before collecting any research data.

Results

For peer review only - http://bmjopen.bmj.com/site/about/guidelines.xhtml

General WISN findings across levels

For descriptive purposes, we have categorized the workload pressure as Extremely High (WISN Ratio between 0.10 and 0.29), Very High (WISN Ratio between 0.30 and 0.49), High (WISN Ratio between 0.50 and 0.69), Moderately High (WISN Ratio between 0.70 and 0.89), Normal (WISN Ratio between 0.90 and 1.19), and Low (WISN Ratio greater than or equal to 1.20). Based on this categorization, at an aggregate level (i.e., considering the average required number and WISN Ratio across the same types of health facilities), physicians are found to have a Very High (WISN Ratio 0.43) and nurses High (WISN Ratio 0.69) workload pressure. To cope with the workload, on an average 11 physicians (on average 4.50 were available during data collection) and 19 nurses (On average 12.75 were available during data collection) are needed in each UpHC (Table 3). This means, there was an average deficit of six members in each staff categories.

Table 3 Analysis of WISN results at aggregate level (average required number and WISN Ratio across same types of health facilities)

Staff category	Required staff to cope with the demand	Average number of existing staff	Deficit of staff	Average WISN Ratio	Workload pressure
Physician	10.59	4.50	6.09	0.43	Very high
Nurse	18.86	12.75	6.11	0.69	High

For peer review only - http://bmjopen.bmj.com/site/about/guidelines.xhtml

Tabulating the total percentage of time spent on all support activities (i.e., category allowance standards) by different staff categories, we found that, 50% of nurses' time are occupied with support activities (Table 4).

Table 4 Comparison of Support Activities across staff categories

Staff category	Total % of support activities
Physician	24%
Nurse	50%

For peer review only

WISN results disaggregated by UpHCs

The required number of staff ranges from eight to 12 among Physicians, and 16 to 23 among Nurses. Highest shortage is observed in Nurses of Sreemangal UpHC (-8.46), followed by Physicians of Kulaura UpHC (-8.28). Workload pressure is the highest among Physicians of Kotchandpur UpHC (WISN Ratio 0.28) and lowest among Nurses of Shailkupa (WISN Ratio 0.87) (Table 5).

Table 5 Analysis of WISN results of Upazila level health staff

Health facility	Current number of staff	Required number, based on WISN	Shortage or excess	WISN Ratio	Workload pressure
**Staff category: Physician**					

Shailkupa UpHC	4	8.14	-4.14	0.49	Very High
Kotchandpur UpHC	3	10.71	-7.71	0.28	Extremely High
Kulaura UpHC	4	12.28	-8.28	0.33	Very High
Sreemangal UpHC	7	11.23	-4.23	0.62	High
**Staff category: Nurse**					
Shailkupa UpHC	14	16.08	-2.08	0.87	Moderately High
Kotchandpur UpHC	15	22.80	-7.8	0.66	High
Kulaura UpHC	10	16.08	-6.08	0.62	High
Sreemangal UpHC	12	20.46	-8.46	0.59	High

Change of workload if vacancies are filled

If the vacant posts are filled, understandably, the workload is reduced. In most of the cases, sanctioned number of Physicians and Nurses is more than what is actually needed to tackle the workload. However, only filling up the vacant posts are not enough in case of some of the staff categories, such as the Nurses at Kotchandpur and Physicians at Sreemangal (Table 6).

For peer review only - http://bmjopen.bmj.com/site/about/guidelines.xhtml

Table 6 Change of workload if vacancies in Physician and Nursing posts are filled

Health facility	Staff category	Current number of staff	Required number, based on WISN	WISN Ratio	Sanctioned number of staff	WISN Ratio as per sanctioned number of staff
Shailkupa UpHC	Physician	4	8.14	0.49	10	1.25
	Nurse	14	16.08	0.87	21	1.31
Kotchandpur UpHC	Physician	3	10.71	0.28	20	1.82
	Nurse	15	22.80	0.66	20	0.87
Kulaura UpHC	Physician	4	12.28	0.33	20	1.67
	Nurse	10	16.08	0.62	26	1.62
Sreemangal UpHC	Physician	7	11.23	0.62	10	0.91
	Nurse	12	20.46	0.59	22	1.10

For peer review only

Discussion and recommendations

Discussion

Findings from this WISN study clearly indicates that the public-sector healthcare providers in Bangladesh are suffering from a very high workload pressure. Nurses are predominantly occupied with support activities rather than actual nursing care. There is unequal workload across UpHCs, indicating potential for workforce re-distribution. The unequal workload mainly stems from

For peer review only - http://bmjopen.bmj.com/site/about/guidelines.xhtml

differing patient load, due to geographic location, number of catchment population, and epidemiological characteristics, at different UpHCs. Inappropriate number of sanctioned posts indicate the necessity of WISN-based workforce planning.

High workload pressure may arise from absolute or relative shortage of health workforce. Absolute shortage appears when there is inadequate production of a particular staff category while relative shortage appears when health workforce is not distributed evenly between the urban and rural areas throughout the country for various reasons. For example, absolute shortage in HRH production is revealed by the fact that there are only 4.90 registered physicians and 2.90 registered nurses per 10,000 populations[18], rendering the country to be one of the 57 critical workforce shortage countries in the world[6]. On top of this absolute shortage, Bangladesh also suffers from relative shortage, as evidenced from the fact that physician to population ratio in urban areas is 1:1,500, but in rural areas it is 1:15,000[21]. Workload pressure has some serious consequences as well, namely, fatigue and burnout of service providers, lack of motivation, and compromised quality of care[22]. High workload is, however, not unique to Bangladesh. WISN studies in Low- and Middle-Income Countries (LMICs) like Namibia[23], Uganda[24], Kenya[25], Burkina Faso[26], and Iran[27] also identified high workload pressure among their HRH.

It is expected that the nurses would spend most of their service times beside the patients, providing nursing care. Unfortunately, this is not the case in Bangladesh as well as in some other comparable settings. A qualitative study in Bangladesh showed, nurses' maximum time is spent on administrative and paperwork tasks[28]. Excessive support activities of nurses is reported in studies conducted in Iran[27], and Uganda[24] as well. A recent WISN study conducted in Iran showed, nurses

For peer review only - http://bmjopen.bmj.com/site/about/guidelines.xhtml

are overburdened; and support activities account for 31% of their workload[27]. Nurses' excessive engagement in paperwork or other support activities may result from deficient human resource planning and management.

Despite the fact that most of the staff are already overworked, staffs in some health facilities may be more so, compared to a neighboring one. Presence of different number of staffs causes fluctuation in the amount of workload at different health facilities. In places where workload of a staff category is 'Extremely High', some supports from nearby health facilities with lower workload should be sought. Or, in places where workload of a staff category is 'Normal' or 'Low', some support may be transferred to health facilities with higher workload. For example, in Sreemangal UpHC, there are seven physicians, with a High workload pressure. However, workload pressure in nearby Kulaura UpHC is Very High, with only four physicians (Table 5). At least one physician from Sreemangal can be reallocated to Kulaura to tackle the high workload. Similar action may be taken regarding the Nurses by transferring some from Shailkupa (Moderately High workload) to Kotchandpur (High workload). This is just an example how WISN can help in decision-making regarding allocation of human resources. Similar situation was identified in Namibia, where researchers suggested redistribution of health workers from one area to the other[23].

We found that many posts remained vacant in different health facilities. Some staffs were not present at their service locations for various reasons, such as training, deputation to another health facility, etc. Even if the existing posts are filled-up, a large portion of the workload would be curbed. For example, according to the Standard Setup document of the Ministry of Public

For peer review only - http://bmjopen.bmj.com/site/about/guidelines.xhtml

Administration, 18 physician posts (10 Junior Consultants, one Residential Medical Officer, seven Assistant Surgeons) have been proposed for a 50 bed hospital[29]. We have found 4.5 Physicians on an average in each UpHC. The average required number is 11 (Table 3). Our proposition is that, even if it is not possible to reach the ideal workforce setup for a health facility, filling-up at least the vacant positions, and ensuring regular presence of all staffs would reduce the workload. Supportive supervision and monitoring of the staff is essential to ensure the presence of posted staff. Researchers in Namibia came up with the similar finding and proposed a similar solution for the problem[23]. WISN was used over standard staffing schedule in HIV Clinics in Kenya as well to resolve a similar crisis[25].

Recommendations

Based on the findings and its in-depth analysis, we propose few short-term and long-term recommendations. The short-term recommendations require administrative or management decisions, relatively easier to implement and tackle the immediate crisis. On the other hand, the long-term recommendations demand radical policy amendments following careful examination.

Short-term recommendations include: reallocation of staff from low workload areas to high workload areas, fill-up existing vacant positions and strengthen supervision and monitoring. Nurses are the most needed staff, the most overloaded, and are short in supply. On top of all these, they are burdened with support activities. If some of their support and additional activities can be shifted to other staff, nurses can devote their time better in nursing care.

For peer review only - http://bmjopen.bmj.com/site/about/guidelines.xhtml

439 The study yields some long-term recommendations as well, for the policy-makers. For example, in order to increase the availability of workforce, especially Nurses, and decrease their workload, their number needs to increase. Hence, long term policy response is needed to increase the intake of nursing students, train them with quality education, and deploy them in larger numbers in a secure and gender-friendly work environment. In the same vein, incentives should be given to increase the number of nurses in both public and private sector educational institutions. Regulations should be developed and implemented so that medical colleges can be established only when a nursing school is established alongside. Otherwise, the skill-mix imbalance between physicians and nurses would jeopardize the quality of care. Quality and quantity of physicians should also increase.

449 Secondly, since the Nurses are found to be predominantly engaged in support activities at the expense of actual patient care, a separate staff category for administrative/ support activities is greatly warranted. This will free up the valuable yet scarce clinical time of the service providers.

452 Thirdly, instead of the existing approach of deploying a fixed number of workforce at all health facilities, a flexible recruitment and HRH planning is needed, based on patient load and disease burden. This can be supported by determination of absolute requirement of HRH in those health facilities by using the WHO methodology on workload indicators of staffing need. It is important to recognize that, decisions in health sector are very much contingent on the local context, especially the patient load, demographic drivers (e.g., age structure of the population, gender ratio, etc.), and epidemiologic profile. Therefore, the government should adopt flexible health workforce planning and recruitment policy in place to keep up with the local patient load and disease burden. The culture of bottom-up decision-making should be adopted eventually.

For peer review only - http://bmjopen.bmj.com/site/about/guidelines.xhtml

Strengths and limitations

This study had a number of strengths. First, we conducted time-motion study, which helped the research team gain a better understanding of the service context of the staffs. Secondly, when the key informants or experts suggested an unrealistic activity standard, we presented them the time-motion findings and helped them suggest a more context sensitive standards. Thirdly, the research team used both qualitative and quantitative methods for primary data collection, which complement each other for bringing data accuracy. Fourthly, WHO technical officers, who had expertise in WISN application in other countries, and the Ministry of Health officials were directly involved in the field level data quality checks.

However, despite careful planning and painstaking implementation of the research, we faced some challenges during different stages of the WISN process. First, some service statistics data, which were essential for establishing standard workloads, were not readily available due to poor record keeping systems at some health facilities. Secondly, the research did not take into account the patients' opinion or stakeholders' stance. Notwithstanding the fact that these perspectives are gaining momentum in health workforce decision-making, we could not take advantage of them in the interest of adhering to the highly structured nature of the WISN methodology. Thirdly, the official number of existing staffs often did not match with the number of staffs we observed providing services.

Conclusions

Human resource management is a big challenge, especially in a resource-poor setting like Bangladesh. With a vision of becoming a middle-income country by 2021, Bangladesh needs to

For peer review only - http://bmjopen.bmj.com/site/about/guidelines.xhtml

strive for optimizing its existing resources, including human resources. This type of study can aid the decision-making in this direction, using the WISN as a planning tool for the managers. Implementation research is needed regarding how this workload-based staffing decisions can be integrated into the health systems in the most effective way. We expect that, these types of studies would pave the way for evidence-based HRH decision-making in the context of health system of Bangladesh.

List of abbreviations

ANC: Antenatal Care;

EMO: Emergency Medical Officer;

ERC: Ethical Review Committee;

ESP: Essential Services Package;

EWG: Expert Working Groups;

FDCs: Field Data Collectors;

FSs: Field Supervisors;

GoB: Government of Bangladesh;

HRH: Human Resources for Heath;

IDI: In-depth Interviews;

IMCI: Integrated Management of Childhood Illness;

KII: Key Informant Interviews;

LMICs: Low- and Middle-Income Countries;

MO: Medical Officer;

MoHFW: Ministry of Health and Family Welfare;

For peer review only

NCD: Non-communicable Diseases;

OPD: Out Patient Department;

PNC: Postnatal Care;

RMO: Residential Medical Officer;

SC: Steering Committee;

TT: Technical Taskforce;

UHC: Universal Health Coverage;

UpHCs: Upazila Health Complexes;

WISN: Workload Indicators of Staffing Need

Declarations

Consent for publication

During the data collection process, while the ethical consents were obtained from the respondents, they were informed that their data might be used for publication in future. They were also informed that their identity will remain anonymous. Institutional consents for publication were obtained as well.

Availability of data and materials

As our study is not involved in developing new software, or new database, therefore, the datasets generated and analyzed during this study (which was developed using WISN software) are not publicly available. But if necessary, we will share our database for future research purposes.

For peer review only - http://bmjopen.bmj.com/site/about/guidelines.xhtml

Competing interests

The authors declared that they do not have any competing interests.

Funding

This study was funded by WHO Country Office, Bangladesh and conducted by a group of researchers at BRAC James P Grant School of Public Health, BRAC University.

Authors' contributions

TJ conceived and designed the study. TJ and SNBKT carried out the data analyses and drafted the manuscript. MN, SA, VOC, and TZ thoroughly reviewed the manuscript and contributed substantially with necessary revision. TJ and SNBKT again reviewed the manuscript and prepared for final submission. All authors approved the final manuscript.

Acknowledgements

BRAC James P Grant School of Public Health, BRAC University acknowledges with gratitude to WHO Country Office, Bangladesh for its contribution in this research effort and MoHFW for supporting the research team throughout the study period. We are also thankful to the study participants who consented to participate in the study. We are indebted to Professor. Dr. Sayed Masud Ahmed, who served as the Advisor to the study project, and provided valuable technical inputs at different stages of the project. Finally, we would like to extend our sincere gratitude to the Field Supervisors and Data Collectors for their contribution throughout the qualitative and quantitative data collection.

For peer review only - http://bmjopen.bmj.com/site/about/guidelines.xhtml

Patient and Public Involvement statement: No patient involved

References

1. International Labor Organization. Global Extension of Social Security. Geneva; 2013.

2. World Health Organization. Global strategy on human resources for health: workforce 2030. Geneva; 2016.

3. WHO South East Asia Regional Office. Decade for health workforce strengthening in the South-East Asia Region 2015-2024, second review of progress; New Delhi; 2018 http://apps.searo.who.int/PDS_DOCS/B5439.pdf

4. Lerberghe WV, Conceic CC, Damme WV, and Ferrinho P. When staff is underpaid : dealing with the individual coping strategies of health personnel. 2002; 80 (01): p. 581–584. https://www.ncbi.nlm.nih.gov/pmc/articles/PMC2567566/pdf/12163923.pdf.

5. Rowe AK, De Savigny D, Lanata CF, Victora CG. How can we achieve and maintain high-quality performance of health workers in low-resource settings?. *The Lancet*. 2005 Sep 17;366: 9490.p.1026-35. https://www.thelancet.com/journals/lancet/article/PIIS0140-6736(05)67028-6/fulltext?code=lancet-site.

6. World Health Organization. The world health report: working together for health. Geneva: World Health Organization; 2006.

7. Henderson LN, Tulloch J. Incentives for retaining and motivating health workers in Pacific and Asian countries. *Human resources for health*. 2008 Dec;6(1):18. doi: 10.1186/1478-4491-6-18.

8. BanglaPedia. National Encyclopedia of Bangladesh. Available at http://en.banglapedia.org/index.php?title=Season (Accessed on 21 August 2019)

For peer review only

For peer review only - http://bmjopen.bmj.com/site/about/guidelines.xhtml

9. Ahmed SM, Evans TG, Standing H, Mahmud S. Harnessing pluralism for better health in Bangladesh. *The Lancet*. 2013 Nov 23;382(9906):1746-55.doi: 10.1016/S0140-6736(13)62147-9.

10. Ahmed SM, Alam BB, Anwar A, Begum T, Haque R, AM Khan J and et al. Bangladesh Health System Review. Edited by A. Naheed and K. Hort. Dhaka: World Health Organization; 2015

11. Cometto G, Witter S. Tackling health workforce challenges to universal health coverage: setting targets and measuring progress. Bulletin of the World Health Organization. 2013;91:881-5. doi: 10.2471/BLT.13.118810.

12. Government of Bangladesh (2015) Bangladesh Health Workforce Strategy 2015. Dhaka.

13. Darkwa EK, Newman MS, Kawkab M, Chowdhury ME. A qualitative study of factors influencing retention of doctors and nurses at rural healthcare facilities in Bangladesh. *BMC health services research.* 2015 Dec;15(1):344. doi: 10.1186/s12913-015-1012-z.

14. Rawal LB, Joarder T, Islam SM, Uddin A, Ahmed SM. Developing effective policy strategies to retain health workers in rural Bangladesh: a policy analysis. *Human resources for health*. 2015 Dec;13(1):36. Available at: http://www.coe-uhc.org/index.php/publications/category/9-working-paper-series.

15. deVaron Reynolds J, Costello T, Edwards MT. The study of workload in child protective and child welfare services. Time and Effort: Perspectives on Workload Roundtable. 2008 Dec 3. Available at: https://www.researchgate.net/profile/Robin_Perry3/publication/237430886_A_Critical_Appraisal_of_What_Child_Welfare_Workers_Do_Findings_From_a_Task_Analysis_Study_in_Florida/links/54662f200cf25b85d17f5b5b.pdf#page=4.

16. World Health Organization. Global strategy on human resources for health: workforce 2030. 2016. Available at: http://apps.who.int/iris/bitstream/10665/250368/1/9789241511131-eng.pdf?ua=1.

For peer review only

For peer review only - http://bmjopen.bmj.com/site/about/guidelines.xhtml

17. World Health Organization (2010) Workload Indicators of Staffing Need: User's Manual. Geneva: World Health Organization.

World Health Organization. Workload indicators of staffing need: User's manual. Geneva: World Health Organization. 2010.

18. Lopetegui M, Yen PY, Lai A, Jeffries J, Embi P, Payne P. Time motion studies in healthcare: what are we talking about?. *Journal of biomedical informatics*. 2014 Jun 1;49:292-9. doi: 10.1016/j.jbi.2014.02.017.

19. Government of Bangladesh. *Health Bulletin*. Dhaka. 2016a.

20. Ritchie J, Lewis J, Nicholls CM, Ormston R, editors. Qualitative research practice: A guide for social science students and researchers. sage; 2013 Nov 1.

21. Government of Bangladesh. Bangladesh Essential Health Service Package (ESP). Dhaka. 2016b.

22. Ahmed SM, Hossain MA, RajaChowdhury AM, Bhuiya AU. The health workforce crisis in Bangladesh: shortage, inappropriate skill-mix and inequitable distribution. Human resources for health. 2011 Dec;9(1):3. Available at: http://www.human-resources-health.com/content/9/1/3.

23. Greenglass ER, Burke RJ, Moore KA. Reactions to increased workload: Effects on professional efficacy of nurses. Applied psychology. 2003 Oct;52(4):580-97. doi: 10.1111/1464-0597.00152.

24. McQuide PA, Kolehmainen-Aitken RL, Forster N. Applying the workload indicators of staffing need (WISN) method in Namibia: challenges and implications for human resources for health policy. *Human resources for health*. 2013 Dec;11(1):64. doi: 10.1186/1478-4491-11-64.

25. Namaganda G, Oketcho V, Maniple E, Viadro C. Making the transition to workload-based staffing: using the Workload Indicators of Staffing Need method in Uganda. *Human resources for health*. 2015 Dec;13(1):89.. doi: 10.1186/s12960-015-0066-7.

26. Burmen B, Owuor N, Mitei P. An assessment of staffing needs at a HIV clinic in a Western Kenya

For peer review only

For peer review only - http://bmjopen.bmj.com/site/about/guidelines.xhtml

using the WHO workload indicators of staffing need WISN, 2011. *Human resources for health*. 2017 Dec;15(1):9. doi: 10.1186/s12960-017-0186-3.

27. Ly A, Kouanda S, Ridde V. Nursing and midwife staffing needs in maternity wards in Burkina Faso referral hospitals. *Human resources for Health*. 2014 May;12(1):S8. doi: 10.1186/1478-4491-12-S1-S8.

28. Nayebi BA, Mohebbifar R, Azimian J, Rafiei S. Estimating nursing staff requirement in an emergency department of a general training hospital: Application of Workload Indicators of Staffing Need (WISN). *International Journal of Healthcare Management*. 2017 Oct 17:1-6. doi: 10.1080/20479700.2017.1390182.

29. Zaman S. Ladies without lamps: nurses in Bangladesh. Qualitative Health Research. 2009 Mar;19(3):366-74. doi: 10.1177/1049732309331876.

30. Ministry of Public Administration. 'Standard setup by the government for human resources in 10, 20, 31, 50, 100, 150, 200, 250, 500, 500 bed general hospitals at Union, Upazila, District, and Division levels, under Ministry of Health and Family Welfare'. Bangladesh. 2008.

For peer review only

Figure 1

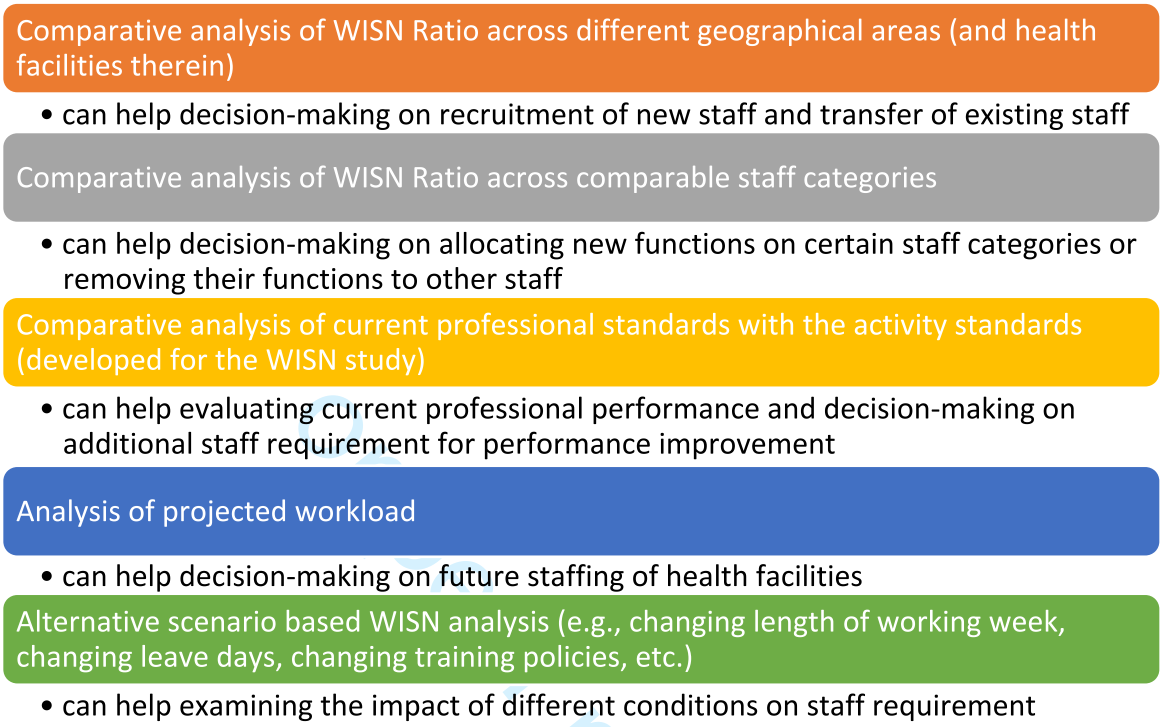

Figure 1. The ways WISN can help in human resource decision-making

For peer review only

Figure 2

Determining priority cadre(s) and health facility types(s)
Priority cadres and facilities (from both communities and facilities) were determined after discussing with the Steering Committee members.

↓

Estimating available working time
Was decided based on the findings from the document reviews, key informant interviews and in-depth interviews.

↓

Defining workload components
Was determined based on key informant interviews with the experts, supplemented by observations and in-depth interviews with active service providers.

↓

Setting activity standards
These data were collected through both in-depth interviews and a quantitative technique, time motion study.

↓

Establishing standard workloads
A standard workload is the amount of work within a health service workload component that one health worker can do in a year. This was calculated using WISN software.

↓

Calculating allowance factors
This is to document the additional and support activities performed by a health staff. This was also calculated using the WISN software.

↓

Determining staff requirement based on WISN
Through secondary data extraction from health facility records, annual service statistics were collected in order to determine the staff requirement

↓

Analyzing and interpreting WISN results

- Comparing the difference between current and required staffing levels, we identified the health facilities that are relatively understaffed or overstaffed
- Using the WISN ratio, we assessed the work pressure that health workers experience

Figure 1 Methods applied in each WISN step

For peer review only - http://bmjopen.bmj.com/site/about/guidelines.xhtml

Figure 3

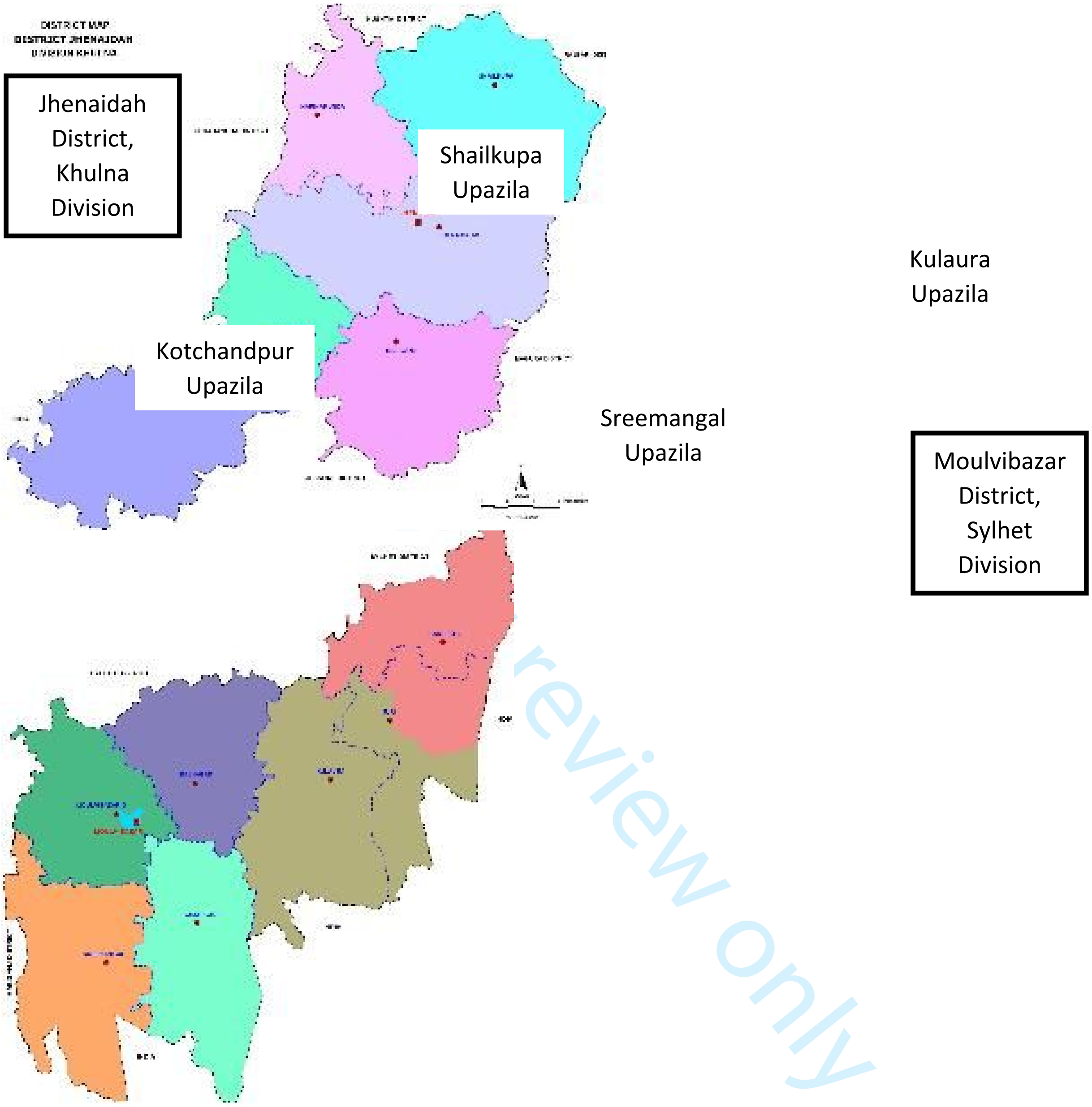

Figure 1 Map of Jhenaidah and Moulvibazar Districts with study Upazilas identified

Figure 4

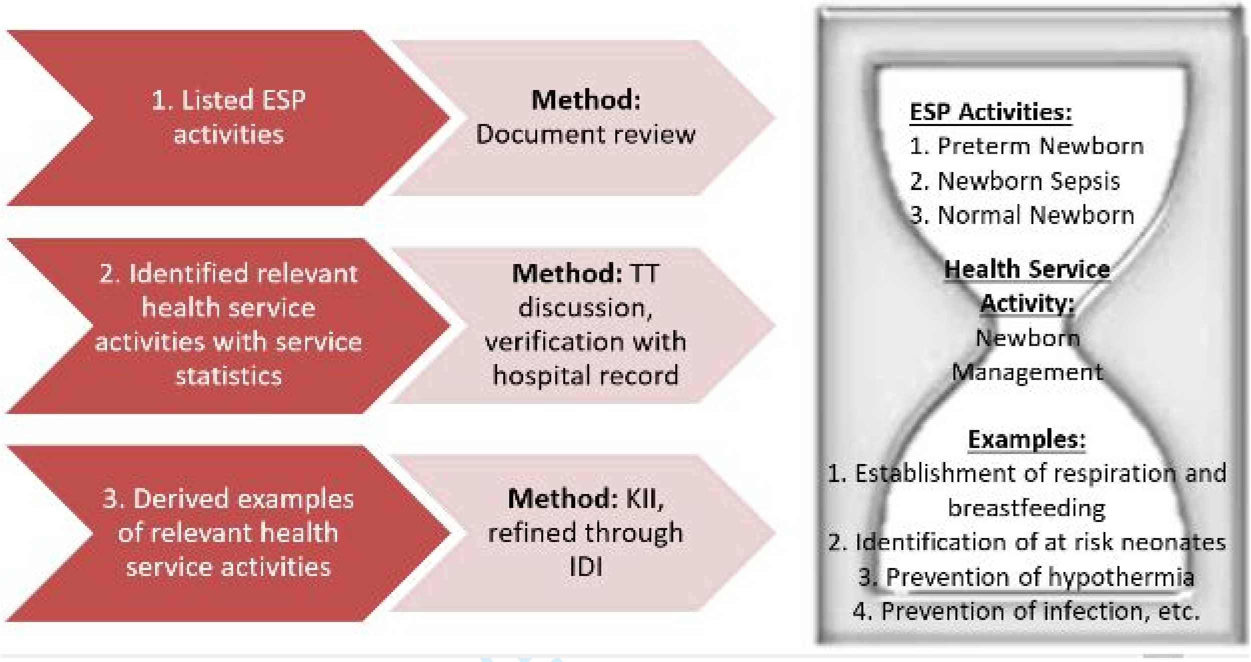

Figure 1 Approach of integration of ESP components in defining workload components of health service activity

An assessment of staffing needs for physicians and nurses at Upazila Health Complexes in Bangladesh using WHO workload indicators of staffing need (WISN) method

Journal:	*BMJ Open*
Manuscript ID	bmjopen-2019-035183.R1
Article Type:	Original research
Date Submitted by the Author:	15-Dec-2019
Complete List of Authors:	Joarder, Taufique; BRAC University, BRAC James P Grant School of Public Health; FHI 360, Bangladesh Office<br>Tune, Samiun; BRAC James P Grant School of Public Health, BRAC University, Public Health;<br>Nuruzzaman, Md; World Health Organization Bangladesh<br>Alam, Sabina; Government of Bangladesh Ministry of Health and Family Welfare, Health Services Division<br>Cruz, Valeria; World Health Organization, Country Office for Bangladesh<br>Zapata, Tomas; SEARO
**Primary Subject Heading**:	Health services research
Secondary Subject Heading:	Health policy, Global health, Nursing, Public health
Keywords:	Health policy < HEALTH SERVICES ADMINISTRATION & MANAGEMENT, PUBLIC HEALTH, HEALTH SERVICES ADMINISTRATION & MANAGEMENT, Human resource management < HEALTH SERVICES ADMINISTRATION & MANAGEMENT

SCHOLARONE™
Manuscripts

I, the Submitting Author has the right to grant and does grant on behalf of all authors of the Work (as defined in the below author licence), an exclusive licence and/or a non-exclusive licence for contributions from authors who are: i) UK Crown employees; ii) where BMJ has agreed a CC-BY licence shall apply, and/or iii) in accordance with the terms applicable for US Federal Government officers or employees acting as part of their official duties; on a worldwide, perpetual, irrevocable, royalty-free basis to BMJ Publishing Group Ltd ("BMJ") its licensees and where the relevant Journal is co-owned by BMJ to the co-owners of the Journal, to publish the Work in this journal and any other BMJ products and to exploit all rights, as set out in our licence.

The Submitting Author accepts and understands that any supply made under these terms is made by BMJ to the Submitting Author unless you are acting as an employee on behalf of your employer or a postgraduate student of an affiliated institution which is paying any applicable article publishing charge ("APC") for Open Access articles. Where the Submitting Author wishes to make the Work available on an Open Access basis (and intends to pay the relevant APC), the terms of reuse of such Open Access shall be governed by a Creative Commons licence – details of these licences and which Creative Commons licence will apply to this Work are set out in our licence referred to above.

Other than as permitted in any relevant BMJ Author's Self Archiving Policies, I confirm this Work has not been accepted for publication elsewhere, is not being considered for publication elsewhere and does not duplicate material already published. I confirm all authors consent to publication of this Work and authorise the granting of this licence.

For peer review only

**Title page

An assessment of staffing needs for physicians and nurses at Upazila Health Complexes in Bangladesh using WHO workload indicators of staffing need (WISN) method

Taufique Joarder[1, 2], Samiun Nazrin Bente Kamal Tune[1*], Md Nuruzzaman[3], Sabina Alam[4], Valeria de Oliveira Cruz[5], Tomas Zapata[5]

[1]BRAC James P Grant School of Public Health, BRAC University, Dhaka

[2]FHI360, Dhaka, Bangladesh

[3]World Health Organization Country Office for Bangladesh, Dhaka

[4]Health Services Division, Ministry of Health and Family Welfare, Government of Bangladesh

[5]World Health Organization South-East Asia Regional Office, New Delhi

Email addresses:

TJ: taufiquejoarder@gmail.com

SNBKT: samiun.tune@gmail.com

MN: nuruzzamanm@who.int

SA: alam6350@gmail.com

VOC: deoliveiracruzv@who.int

TZ: zapatat@who.int

*Corresponding author

For peer review only - http://bmjopen.bmj.com/site/about/guidelines.xhtml

Abstract

Objective This study aimed to assess the current workload and staffing need of Physicians and Nurses for delivering optimum health care services at the Upazila Health Complexes (UpHCs) in Bangladesh.

Design Mixed-methods, combining qualitative (e.g., document reviews, key informant interviews, in-depth interviews, observations) and quantitative methods (time-motion survey)

Setting Study was conducted in 24 health facilities of Bangladesh. However, UpHCs being the nucleus of primary health care in Bangladesh, this manuscript limits itself to reporting the findings from the providers at four UpHCs under this project.

Participants 18 Physicians and 51 Nurses, males and females.

Primary outcome measures Workload components were defined based on inputs from five experts, refined by nine service providers. Using WHO WISN software, standard workload, category allowance factor, individual allowance factor, total required number of staff, WISN Difference, and WISN Ratio were calculated.

Results: Physicians have Very High (WISN Ratio 0.43) and Nurse High (WISN Ratio 0.69) workload pressure. 50% of Nurses' time are occupied with support activities, instead of nursing care. There are different workloads among the same staff category in different health facilities. If only the vacant posts are filled, the workload is reduced. In fact, sanctioned number of Physicians and Nurses is more than actual need.

Conclusions: It is evident that high workload pressures prevail for Physicians and Nurses at the UpHCs. This reveals high demand for these health workforces in the respective sub-districts. WISN method can aid the policy-makers in optimizing utilization of existing human resources. Therefore, the government should adopt flexible health workforce planning and recruitment policy

For peer review only - http://bmjopen.bmj.com/site/about/guidelines.xhtml

to manage the patient load and disease burden. WISN should thus be incorporated as a planning tool for health managers. There should be a regular review of health workforce management decisions, and these should be amended based on periodic reviews.

Strengths and limitations

- Time-motion findings helped the experts suggest a more context sensitive activity standards.
- Using both qualitative and quantitative methods for primary data collection complemented each other for bringing data accuracy.
- Technical inputs from the WHO technical experts in WISN application in other countries, and officials of the Ministry of Health improved the data quality as they were directly involved in quality checks at the field level.
- One limitation was that some service statistics data, essential for establishing standard workloads, were unavailable.
- Due to lack of scope in WISN methodology, patient engagement was minimal.

Keywords

Human Resources for Health, Workforce Management, Workload Indicators of Staffing Need, Bangladesh, Upazila Health Complex, Health Systems

For peer review only - http://bmjopen.bmj.com/site/about/guidelines.xhtml

Background

Shortage of Human Resources for Heath (HRH) has been one of the major challenges faced by the health system and globally, more than 90 countries are haunted by this crisis. According to International Labor Organization (ILO), there are on average 34.5 health workers per 10,000 population and about one third of the world's population lack access to health care because of shortage of health workforce[1]. According to the Global strategy on human resources for health: workforce 2030, the estimated global shortage of skilled health workers will be around 18 million by 2030[2]. This problem has reached a critical stage in three (Bangladesh, Myanmar and Bhutan) South-East Asia Region countries with <23 health workers (doctors, nurses and midwives) per 10,000 population, limiting access to health services[3].

Improving health workers' performance and productivity is vital for better health service provision in the country. Poor performance of the health workers has been reported in the literature resulting from too few staff, or staff not providing care according to standards[4, 5, 6]. The extent of the shortage is reflected in health worker density rates and workforce vacancy rates, and its impact in health system performance indicators. Factors that contribute to poor performance of health workers include limited employment opportunities and low salaries; poor working conditions, weak support and supervision, and limited opportunities for professional development[7].

Bangladesh's health workforce scenario is characterized by "shortage, inappropriate skill mix and inequitable distribution"[8, 9]. Equitable access to skilled and motivated health worker in a functional health system is essential for achieving Universal Health Coverage (UHC) and the Sustainable Development Goals[10]. In 2015, Government of Bangladesh (GoB) approved the Bangladesh

For peer review only - http://bmjopen.bmj.com/site/about/guidelines.xhtml

Health Workforce Strategy which affirms government's vision of equitable availability of skilled, motivated and responsive health workforce in adequate numbers across the country[11]. However, there is lack of comprehensive, nationally representative data on HRH workload and optimum staff need in health care facilities in Bangladesh. A small scale qualitative study found overwhelming workload as one of the critical components that hinders retention of doctors and nurses at rural healthcare facilities in Bangladesh[12]. Another policy analysis on retention of HRH (physicians and nurses) also found that deficiency of adequate workforce and consequent high workload acted as a deterrent against rural retention[13].

Workload management is very important for any country or institution to deliver quality services, retain staffs and reduce turnover[14]. Even the seminal document on HRH, "Global strategy on human resources for health: Workforce 2030", emphasized on developing country level workforce strategies, drawing on workload analysis studies[15]. Such studies can provide detailed insight into the current state of workload in a system, coping strategies of the staff for regular extra work pressure, causes behind the excessive workloads, and ways to deal with it. This study aimed to fill-in this knowledge gap with respect to workload and optimum staff need for physicians and nurses at the Upazila or Sub-district level (i.e., at Upazila Health Complex [UpHC]). It is expected that this workload analysis will contribute in improving performance, ensuring quality of services, and facilitating uninterrupted service delivery through efficient management of staff.

Workload Indicator of Staffing Need (WISN) overview

WHO developed the Workload Indicator of Staffing Need (WISN) method in 1998, which was later updated based on learning from implementation in different countries. This method is simple,

For peer review only - http://bmjopen.bmj.com/site/about/guidelines.xhtml

useful and time-saving, which was borrowed from the industrial sector for use in the health sector by Peter Shipp in 1984. The result is expressed in terms of differences and ratios, the former indicating worker shortage or surplus, and the latter workload pressure experienced by the staff [16].

WISN results help in human resource decision-making in several ways (Figure 1). For example, recruitment and transfer of HRH can be based on geographic comparison of WISN ratios, staffing of health facilities can be informed by WISN-based workload projection, etc.

Methods

Study design

We followed the updated WISN manual[16], but contextualized it for Bangladeshi setting. The WISN steps have been summarized in Figure 2. The research project was developed based on close collaboration among and mutual insights from three types of committees:

1. **Steering Committee (SC):** The SC was consisting 13 members, established by the Ministry of Health and Family Welfare (MOHFW) with membership from senior government officials (seven): WHO official (one); professional organization of the physicians, Bangladesh Medical Association (one); and relevant academia (four) such as BRAC University (two persons), Bangladesh University of Health Sciences, and Center for Medical Education. All seven senior government officials were directly involved in decision making regarding daily management of the health workforce in their respective departments. WHO officer was there to respond to WISN related technical issues and application. All four academicians were part of the committee because they had expertise in their respective areas (i.e. education, policy making, curriculum development and performance assessment) of the health workforce. They were also

For peer review only - http://bmjopen.bmj.com/site/about/guidelines.xhtml

well known in the community of scientific writing and academic teaching. The role of the SC was to guide and endorse the overall study based on the WISN strategy and its implementation.

2. **Technical Taskforce (TT):** TT was responsible for guiding the implementation of the WISN process. Researchers from implementing research institution (the school of public health of a Bangladeshi university, BRAC University); and experts from WHO Bangladesh Country Office; an international non-governmental organization (NGO), Save the Children; and another university, Bangladesh University of Health Sciences served in the Taskforce.
3. **Expert Working Groups (EWG):** There were multiple EWGs, one for each of the following professional groups: General Physicians (Medical Officer [MO], Emergency Medical Officer [EMO], Residential Medical Officer [RMO]), and Nursing Staff (Senior Staff Nurse, Nursing Supervisor). The respective EWG defined the workload components and set activity standard for the specific staff category.

The qualitative part of this research involved document reviews, key informant interviews (KII) with policy level persons related to HRH issues in Bangladesh (mostly from among SC and EWG members), in-depth interviews (IDI) with individual service providers (e.g., physicians, nurses, etc. working in UpHCs under this study), and observations. The quantitative component involved time-motion survey which is a work measurement technique for recording the times and rates of working for the elements of a specific job though observing a subject continuously or in a certain period of time[17]. Time motion data served as a guide to determine the activity standard for WISN analyses.

For peer review only - http://bmjopen.bmj.com/site/about/guidelines.xhtml

Study duration, setting and population

The research continued from July to November 2017 and was carried out in two pre-selected Districts of Bangladesh (Figure 3):

- Jhenaidah, located in south-western part of Bangladesh, under Khulna Division
- Moulvibazar, located in north-eastern part of Bangladesh, under Sylhet Division

The selection of the districts was made by the Human Resources Unit, Ministry of Health and Family Welfare **in collaboration with the development partners (Save the Children and WHO Bangladesh) in a workshop nationally organized in November 2016. Later the selection of the UpHCs was made in consultation with the SC, taking into consideration some performance indicators such as** number of beds, number of total deliveries, number of live births, bed occupancy rates as well as patient load including the number of outpatient visits. **From each district, two highest performing UpHCs were included in the study**[18]. Thus, Shailkupa and Kotchandpur UpHCs were selected from Jhenaidah District and Kulaura and Sreemangal from the Moulvibazar. Because future applications need to be based on optimum quality standards, the highest performing UpHCs were selected to serve as a model for other health facilities. From each of these four UpHCs, physicians (Medical Officers and Residential Medical Officers) and nurses (Senior Staff Nurse and Nursing Supervisor) were included for workload analysis. A total of 24 health facilities from the two districts were studied. This included two district hospitals, four UpHCs, two Maternal and Child Welfare Centers, eight Union Sub Centers, eight Union Health and Family Welfare Centers, and eight Community Clinics. This manuscript reported findings from all the four UpHCs under this study, as UpHCs are the nucleus of the primary health care delivery in Bangladesh, serving the rural population.

For peer review only - http://bmjopen.bmj.com/site/about/guidelines.xhtml

Sampling strategy

Qualitative part

Documents for review were selected based on the suggestions from the experts (members of SC, TT, and EWG), supplemented by reference tracking of government reports and published literature on HRH of Bangladesh. Key Informants were selected on the principles of purposive sampling[19], supplemented by snowball sampling (i.e., based on the reference or suggestion from the key informants). IDI respondents were selected through purposive sampling, based on the respondent's seniority and designation (e.g., Residential Medical Officer, Nursing Supervisors, etc.). These respondents were practicing individuals and had more than 10 years of experience and played a supervisory role in their respective health facilities.

Quantitative part

For time-motion study, time sampling was done for each consenting staff available during the data collection period. Field Data Collectors (FDCs) observed each staff twice for 45-minutes duration, once during the first half of their service duration and again during the second half. This was done to minimize the bias in the time data due to the patient load (assuming higher patient load in the first half and lower in the second).

For peer review only - http://bmjopen.bmj.com/site/about/guidelines.xhtml

Tool development, pretest, training of data collectors, agreement test

For qualitative data collection, semi-structured guidelines, including that for document reviews, KIIs, IDIs, and observation, were developed. For the time-motion study, structured observation tool was designed. The structured observation tools contained three sections:

1. Background information of observation setting and the person under observation
2. Time-motion data sheet (containing three columns: type of activity, time spent in minutes, and remarks)
3. Example of the activities (health service activities, support activities, and additional activities)

Health service activities, according to WISN manual, are performed by all members of the staff category and regular service statistics are available for them for example, obstetrical service, emergency service, outpatient service, etc. Support activities are also performed by all members of the staff category, but regular service statistics are not available for them for example, record keeping and reporting, attending meetings, instrument sterilization, etc. Additional activities are performed by only certain members of the staff category (e.g., the supervisor or a senior member), and regular service statistics are not available for them for example, duty roster preparation, preparing staff evaluation reports, supervision of cleanliness, etc.[17].

The examples of health service activities were primarily drawn from the list of the activities mentioned in the Essential Services Package (ESP)[20] for respective health facility type. Since service statistics were not available according the ESP activity list, it was adjusted for the local context with inputs from the respondents (through KII, followed by IDI), in alignment with the

For peer review only - http://bmjopen.bmj.com/site/about/guidelines.xhtml

availability of service statistics. An ‘hourglass’ approach was adopted for defining the workload components based on the ESP (Figure 4). Tools were pre-tested in a UpHC near Dhaka, before applying for actual data collection. Qualitative tools were also pre-tested through mock IDIs and KIIs. The pre-testing exercise was followed by the training of the Field Supervisors (FSs) and Field Data Collectors (FDCs).

Data collection and quality control

At first, FSs were sent to respective districts to orient the personnel on the project, seek support, and assess the availability of the service statistics. The FSs spent one week in each district and conducted qualitative observation of the service provision at the UpHCs, to gain a firsthand understanding of the context.

In the second step, we conducted five KIIs to define workload components, in light of the ESP document’s standard of services by facility level[20]. The workload components were further refined based on IDIs with a total of eight physicians and eight nurses.

In the third step, the FDCs, under the supervision of FSs, conducted time-motion study, using a mobile device (SurveyCTO software). During the time FDCs were collecting time-motion data, the FS, in addition to supervising the FDCs, conducted additional IDIs and collected data on available working time; time required for health service, support, and additional activities; and service statistics.

For peer review only - http://bmjopen.bmj.com/site/about/guidelines.xhtml

The next step was data validation and set activity standards. Primary data validation was done through phone calls made to the services providers, and health facility statisticians. Secondly, these were shared with the SC and TT members. Finally, interviews were conducted with the EWG members to finalize the activity standards (Table 1).

Table 1 Number of interviewees representing EWG of staff categories

EWG representing staff category	Number
Physicians: MO, EMO, RMO	2
Nurses: Nursing Supervisor, Senior Staff Nurse	7
Total	9

We presented the time-motion findings to the EWG members and requested them to be as realistic as possible in suggesting activity standards. We also requested them to account for the variability of patient load due to factors such as seasonality, timing of day, facility catchment population, etc. Finally, through multiple meetings, debates and deliberations, the activity standard was finalized, taking into account the information from the IDIs and KIIs, and inputs from the EWG members (Table 2).

Table 2 Service standard for Physicians and Nurses in UpHCs, Bangladesh, 2017

Activities	Service standard		Unit
	Physician	Nurse	

For peer review only - http://bmjopen.bmj.com/site/about/guidelines.xhtml

Obstetrical Service (Caesarean Section)[a]	90	90	min/patient
Obstetrical Service (Normal Delivery)	60	120	min/patient
Newborn Management	15	15	min/inpatient day
Emergency Service	15	Not applicable	min/patient
IMCI/ Nutritional Service	15	15	min/patient
OPD Service (including NCD management)	10	Not applicable	min/patient
First ANC	20	20	min/patient
Follow-up ANC	10	10	min/patient
PNC	15	15	min/patient
Indoor Services (Round, including minor bedside procedures)[b]	25.65	34	min/inpatient day
Bedside patient care	Not applicable	17	min/inpatient day
Patient admission and discharge	Not applicable	20	min/inpatient day
Death certification and associated arrangements	20	30	min/patient

256 **Note:** [a]For Nurses: Assist Obstetrical Service (Caesarean Section); [b]For Nurses: Indoor Services
257 (Round with Physician)
258 # IMCI: Integrated Management of Childhood Illness; OPD: Out Patient Department; NCD: Non-
259 communicable Diseases; ANC: Antenatal Care; PNC: Postnatal Care.

For peer review only - http://bmjopen.bmj.com/site/about/guidelines.xhtml

In order to ensure the quality of data, the Principal Expert (lead author of this article), Co-experts (two co-experts – one was leading data collection and the other was leading data quality check and reporting), WHO team consisting of national and international technical experts, and officials from the Human Resource Branch of Ministry of Health and Family Welfare (MoHFW) conducted field visits to each study District and the health facilities therein. During the time-motion data collection period, the Co-experts monitored the data and their geographic location in real-time. They also regularly checked the consistency of the data. Our field based data collection team saved contact information of all the respondents; so, in case of any confusion or need for clarification, the Co-experts called the respondents over phone.

Data management and analysis

The first analytic step was to estimate available working time of the staffs. This is the time a health worker has available in one year to do his or her work, taking into account authorized and unauthorized absences[17]. For all categories of staff, a uniform number of weeks per year (52 weeks), working days in one week (six days), possible working days in one year (52 * 6 = 312 days) were estimated. Next, absent days, such as public holidays (20 days), earned leave (average for each staff category, based on Health Management Information System data), and casual leave (20 days) were deducted to obtain the annual working time in days. Multiplying this with daily working hours (six hours per day), we obtained annual working time in hours.

For peer review only - http://bmjopen.bmj.com/site/about/guidelines.xhtml

Workload components were defined through the inputs from the key informants; activity standards were also set through the interviews with the EWG members. An activity standard is the time necessary for a well-trained, skilled and motivated worker to perform an activity to professional standards in the local circumstances[17]. Both service standards (for health service activities), category allowance standards (for support activities), and individual allowance standards (for additional activities) were determined in the same way.

The next analytic step was to establish standard workload, which was done by dividing the annual working time by unit time of health service activities. A standard workload is the amount of work within a health services workload component that one health service provider can do in a year hypothetically[17]. Then category allowance factor and individual allowance factors were calculated using the following formula, respectively:

Category allowance factor = 1 / {1 – (Total category allowance standard / 100)}

Individual allowance factor = Total individual allowance standard / Available working time in hours

Next, exact number of required staff was calculated by the following formula:

Total required number of staff = (Staff needed for health service activity * Category allowance factor) + Individual allowance factor

The fractional results were rounded up or down, following the guideline provided in the WISN manual[17]:

- 1.0 – 1.1 is rounded down to 1 and >1.1 – 1.9 is rounded up to 2
- 2.0 – 2.2 is rounded down to 2 and >2.2 – 2.9 is rounded up to 3
- 3.0 – 3.3 is rounded down to 3 and >3.3 – 3.9 is rounded up to 4

For peer review only - http://bmjopen.bmj.com/site/about/guidelines.xhtml

- 4.0 – 4.4 is rounded down to 4 and >4.4 – 4.9 is rounded up to 5
- 5.0 – 5.5 is rounded down to 5 and >5.5 – 5.9 is rounded up to 6

Finally, based on the existing number of staff in the respective health facilities, we calculated both the difference (current number of staff – required number of staff by WISN), and the ratio (current number of staff / required number of staff by WISN). The WISN difference indicates whether the health facilities are relatively understaffed (i.e., when the WISN difference is negative), overstaffed (i.e., when the WISN difference is positive), or balanced (i.e., when the WISN difference is zero). The WISN Ratio indicates whether the staffs are experiencing high workload (i.e., when the WISN Ratio is lower than one), low workload (i.e., when the WISN Ratio is higher than one), or normal workload (i.e., when the WISN Ratio is equal to one). For this calculation, we used the de-facto number of current staff, i.e., the number of staff that we actually found working in the health facilities during our data collection period; not the number shown in the office records or statistics.

Ethical considerations

Ethical approval for this study was obtained from the Ethical Review Committee (ERC) of BRAC James P Grant School of Public Health, BRAC University. All ethical principles were strictly adhered to. We obtained a support letter from the Ministry of Health and Family Welfare and also obtained written informed consent form from each person we engaged in data collection. Identity of respondents was kept confidential.

For peer review only - http://bmjopen.bmj.com/site/about/guidelines.xhtml

Results

General WISN findings across levels

For descriptive purposes, we have categorized the workload pressure as Extremely High (WISN Ratio between 0.10 and 0.29), Very High (WISN Ratio between 0.30 and 0.49), High (WISN Ratio between 0.50 and 0.69), Moderately High (WISN Ratio between 0.70 and 0.89), Normal (WISN Ratio between 0.90 and 1.19), and Low (WISN Ratio greater than or equal to 1.20). Based on this categorization, at an aggregate level (i.e., considering the average required number and WISN Ratio across the same types of health facilities), physicians are found to have a Very High (WISN Ratio 0.43) and nurses High (WISN Ratio 0.69) workload pressure. To cope with the workload, on an average 11 physicians (on average 4.50 were available during data collection) and 19 nurses (On average 12.75 were available during data collection) are needed in each UpHC (Table 3). This means, there was an average deficit of six members in each staff categories.

Table 3 Analysis of WISN results at aggregate level (average required number and WISN Ratio across same types of health facilities)

Staff category	Required staff to cope with the demand	Average number of existing staff	Deficit of staff	Average WISN Ratio	Workload pressure
Physician	10.59	4.50	6.09	0.43	Very high
Nurse	18.86	12.75	6.11	0.69	High

For peer review only - http://bmjopen.bmj.com/site/about/guidelines.xhtml

342 Tabulating the total percentage of time spent on all support activities (i.e., category allowance
343 standards) by different staff categories, we found that, 50% of nurses' time are occupied with
344 support activities (Table 4).

345

346 **Table 4 Comparison of Support Activities across staff categories**

Staff category	Total % of support activities
Physician	24%
Nurse	50%

347

348 **WISN results disaggregated by UpHCs**

349 The required number of staff ranges from eight to 12 among Physicians, and 16 to 23 among
350 Nurses. Highest shortage is observed in Nurses of Sreemangal UpHC (-8.46), followed by
351 Physicians of Kulaura UpHC (-8.28). Workload pressure is the highest among Physicians of
352 Kotchandpur UpHC (WISN Ratio 0.28) and lowest among Nurses of Shailkupa (WISN Ratio 0.87)
353 (Table 5).

354

355 **Table 5 Analysis of WISN results of Upazila level health staff**

Health facility	Current number of staff	Required number, based on WISN	Shortage or excess	WISN Ratio	Workload pressure
**Staff category: Physician**					

For peer review only - http://bmjopen.bmj.com/site/about/guidelines.xhtml

Shailkupa UpHC	4	8.14	-4.14	0.49	Very High
Kotchandpur UpHC	3	10.71	-7.71	0.28	Extremely High
Kulaura UpHC	4	12.28	-8.28	0.33	Very High
Sreemangal UpHC	7	11.23	-4.23	0.62	High
**Staff category: Nurse**					
Shailkupa UpHC	14	16.08	-2.08	0.87	Moderately High
Kotchandpur UpHC	15	22.80	-7.8	0.66	High
Kulaura UpHC	10	16.08	-6.08	0.62	High
Sreemangal UpHC	12	20.46	-8.46	0.59	High

Change of workload if vacancies are filled

If the vacant posts are filled, understandably, the workload is reduced. In most of the cases, sanctioned number of Physicians and Nurses is more than what is actually needed to tackle the workload. However, only filling up the vacant posts are not enough in case of some of the staff categories, such as the Nurses at Kotchandpur and Physicians at Sreemangal (Table 6).

For peer review only - http://bmjopen.bmj.com/site/about/guidelines.xhtml

362

363 Table 6 Change of workload if vacancies in Physician and Nursing posts are filled

Health facility	Staff category	Current number of staff	Required number, based on WISN	WISN Ratio	Sanctioned number of staff	WISN Ratio as per sanctioned number of staff
Shailkupa UpHC	Physician	4	8.14	0.49	10	1.25
	Nurse	14	16.08	0.87	21	1.31
Kotchandpur UpHC	Physician	3	10.71	0.28	20	1.82
	Nurse	15	22.80	0.66	20	0.87
Kulaura UpHC	Physician	4	12.28	0.33	20	1.67
	Nurse	10	16.08	0.62	26	1.62
Sreemangal UpHC	Physician	7	11.23	0.62	10	0.91
	Nurse	12	20.46	0.59	22	1.10

364

365 # Discussion and recommendations

366 ## Discussion

367 Findings from this WISN study clearly indicates that the public-sector healthcare providers in
368 Bangladesh are suffering from a very high workload pressure. Nurses are predominantly occupied
369 with support activities rather than actual nursing care. There is unequal workload across UpHCs,
370 indicating potential for workforce re-distribution. The unequal workload mainly stems from

For peer review only - http://bmjopen.bmj.com/site/about/guidelines.xhtml

For peer review only

differing patient load, due to geographic location, number of catchment population, and epidemiological characteristics, at different UpHCs. Inappropriate number of sanctioned posts indicate the necessity of WISN-based workforce planning.

High workload pressure may arise from absolute or relative shortage of health workforce. Absolute shortage appears when there is inadequate production of a particular staff category while relative shortage appears when health workforce is not distributed evenly between the urban and rural areas throughout the country for various reasons. For example, absolute shortage in HRH production is revealed by the fact that there are only 4.90 registered physicians and 2.90 registered nurses per 10,000 populations[18], rendering the country to be one of the 57 critical workforce shortage countries in the world[6]. On top of this absolute shortage, Bangladesh also suffers from relative shortage, as evidenced from the fact that physician to population ratio in urban areas is 1:1,500, but in rural areas it is 1:15,000[21]. Workload pressure has some serious consequences as well, namely, fatigue and burnout of service providers, lack of motivation, and compromised quality of care[22]. High workload is, however, not unique to Bangladesh. WISN studies in Low- and Middle-Income Countries (LMICs) like Namibia[23], Uganda[24], Kenya[25], Burkina Faso[26], and Iran[27] also identified high workload pressure among their HRH.

It is expected that the nurses would spend most of their service times beside the patients, providing nursing care. Unfortunately, this is not the case in Bangladesh as well as in some other comparable settings. A qualitative study in Bangladesh showed, nurses' maximum time is spent on administrative and paperwork tasks[28]. Excessive support activities of nurses is reported in studies conducted in Iran[27], and Uganda[24] as well. A recent WISN study conducted in Iran showed, nurses

For peer review only - http://bmjopen.bmj.com/site/about/guidelines.xhtml

are overburdened; and support activities account for 31% of their workload[27]. Nurses' excessive engagement in paperwork or other support activities may result from deficient human resource planning and management.

Despite the fact that most of the staff are already overworked, staffs in some health facilities may be more so, compared to a neighboring one. Presence of different number of staffs causes fluctuation in the amount of workload at different health facilities. In places where workload of a staff category is 'Extremely High', some supports from nearby health facilities with lower workload should be sought. Or, in places where workload of a staff category is 'Normal' or 'Low', some support may be transferred to health facilities with higher workload. For example, in Sreemangal UpHC, there are seven physicians, with a High workload pressure. However, workload pressure in nearby Kulaura UpHC is Very High, with only four physicians (Table 5). At least one physician from Sreemangal can be reallocated to Kulaura to tackle the high workload. Similar action may be taken regarding the Nurses by transferring some from Shailkupa (Moderately High workload) to Kotchandpur (High workload). This is just an example how WISN can help in decision-making regarding allocation of human resources. Similar situation was identified in Namibia, where researchers suggested redistribution of health workers from one area to the other[23].

We found that many posts remained vacant in different health facilities. Some staffs were not present at their service locations for various reasons, such as training, deputation to another health facility, etc. Even if the existing posts are filled-up, a large portion of the workload would be curbed. For example, according to the Standard Setup document of the Ministry of Public

For peer review only - http://bmjopen.bmj.com/site/about/guidelines.xhtml

Administration, 18 physician posts (10 Junior Consultants, one Residential Medical Officer, seven Assistant Surgeons) have been proposed for a 50 bed hospital[29]. We have found 4.5 Physicians on an average in each UpHC. The average required number is 11 (Table 3). Our proposition is that, even if it is not possible to reach the ideal workforce setup for a health facility, filling-up at least the vacant positions, and ensuring regular presence of all staffs would reduce the workload. Supportive supervision and monitoring of the staff is essential to ensure the presence of posted staff. Researchers in Namibia came up with the similar finding and proposed a similar solution for the problem[23]. WISN was used over standard staffing schedule in HIV Clinics in Kenya as well to resolve a similar crisis[25].

Recommendations

Based on the findings and its in-depth analysis, we propose few short-term and long-term recommendations. The short-term recommendations require administrative or management decisions, relatively easier to implement and tackle the immediate crisis. On the other hand, the long-term recommendations demand radical policy amendments following careful examination.

Short-term recommendations include: reallocation of staff from low workload areas to high workload areas, fill-up existing vacant positions and strengthen supervision and monitoring. Nurses are the most needed staff, the most overloaded, and are short in supply. On top of all these, they are burdened with support activities. If some of their support and additional activities can be shifted to other staff, nurses can devote their time better in nursing care.

For peer review only - http://bmjopen.bmj.com/site/about/guidelines.xhtml

The study yields some long-term recommendations as well, for the policy-makers. For example, in order to increase the availability of workforce, especially Nurses, and decrease their workload, their number needs to increase. Hence, long term policy response is needed to increase the intake of nursing students, train them with quality education, and deploy them in larger numbers in a secure and gender-friendly work environment. In the same vein, incentives should be given to increase the number of nurses in both public and private sector educational institutions. Regulations should be developed and implemented so that medical colleges can be established only when a nursing school is established alongside. Otherwise, the skill-mix imbalance between physicians and nurses would jeopardize the quality of care. Quality and quantity of physicians should also increase.

Secondly, since the Nurses are found to be predominantly engaged in support activities at the expense of actual patient care, a separate staff category for administrative/ support activities is greatly warranted. This will free up the valuable yet scarce clinical time of the service providers.

Thirdly, instead of the existing approach of deploying a fixed number of workforce at all health facilities, a flexible recruitment and HRH planning is needed, based on patient load and disease burden. This can be supported by determination of optimal requirements of HRH under given limited resources or constrains in those health facilities by using the WHO methodology on workload indicators of staffing need. It is important to recognize that, decisions in health sector are very much contingent on the local context, especially the patient load, demographic drivers (e.g., age structure of the population, gender ratio, etc.), and epidemiologic profile. Therefore, the government should adopt flexible health workforce planning and recruitment policy in place to keep up with the local patient load and disease burden. The culture of bottom-up decision-making should be adopted eventually.

For peer review only

For peer review only - http://bmjopen.bmj.com/site/about/guidelines.xhtml

Strengths and limitations

This study had a number of strengths. First, we conducted time-motion study, which helped the research team gain a better understanding of the service context of the staffs. Secondly, when the key informants or experts suggested an unrealistic activity standard, we presented them the time-motion findings and helped them suggest a more context sensitive standards. Thirdly, the research team used both qualitative and quantitative methods for primary data collection, which complement each other for bringing data accuracy. Fourthly, WHO technical officers, who had expertise in WISN application in other countries, and the Ministry of Health officials were directly involved in the field level data quality checks.

However, despite careful planning and painstaking implementation of the research, we faced some challenges during different stages of the WISN process. First, some service statistics data, which were essential for establishing standard workloads, were not readily available due to poor record keeping systems at some health facilities. Secondly, the research did not take into account the patients' opinion or stakeholders' stance. Notwithstanding the fact that these perspectives are gaining momentum in health workforce decision-making, we could not take advantage of them in the interest of adhering to the highly structured nature of the WISN methodology. Thirdly, the official number of existing staffs often did not match with the number of staffs we observed providing services.

Conclusions

For peer review only - http://bmjopen.bmj.com/site/about/guidelines.xhtml

Human resource management is a big challenge, especially in a resource-poor setting like Bangladesh. With a vision of becoming a middle-income country by 2021, Bangladesh needs to strive for optimizing its existing resources, including human resources. This type of study can aid the decision-making in this direction, using the WISN as a planning tool for the managers. Implementation research is needed regarding how this workload-based staffing decisions can be integrated into the health systems in the most effective way. We expect that, these types of studies would pave the way for evidence-based HRH decision-making in the context of health system of Bangladesh.

List of abbreviations

ANC: Antenatal Care;

EMO: Emergency Medical Officer;

ERC: Ethical Review Committee;

ESP: Essential Services Package;

EWG: Expert Working Groups;

FDCs: Field Data Collectors;

FSs: Field Supervisors;

GoB: Government of Bangladesh;

HRH: Human Resources for Heath;

IDI: In-depth Interviews;

IMCI: Integrated Management of Childhood Illness;

KII: Key Informant Interviews;

LMICs: Low- and Middle-Income Countries;

For peer review only - http://bmjopen.bmj.com/site/about/guidelines.xhtml

MO: Medical Officer;

MoHFW: Ministry of Health and Family Welfare;

NCD: Non-communicable Diseases;

OPD: Out Patient Department;

PNC: Postnatal Care;

RMO: Residential Medical Officer;

SC: Steering Committee;

TT: Technical Taskforce;

UHC: Universal Health Coverage;

UpHCs: Upazila Health Complexes;

WISN: Workload Indicators of Staffing Need

Declarations

Consent for publication

During the data collection process, while the ethical consents were obtained from the respondents, they were informed that their data might be used for publication in future. They were also informed that their identity will remain anonymous. Institutional consents for publication were obtained as well.

Availability of data and materials

All research data have been submitted to the World Health Organization Country Office for Bangladesh, as per the agreement with the research organization, BRAC James P Grant School of Public Health, BRAC University. This manuscript only used the data pertaining to the Physicians

For peer review only - http://bmjopen.bmj.com/site/about/guidelines.xhtml

and Nurses working at the two study Upazila (sub-district) Health Complexes. Unpublished data include: Consultants, General Physicians, and Nurses at the District Hospital level; Physicians, and Family Welfare Visitors at Maternal and Child Welfare Center level; Sub-Assistant Community Medical Officers at the Upazila Health Complex level; Sub-Assistant Community Medical Officers at Union Sub-Centers level, Sub-Assistant Community Medical Officers, and Family Welfare Visitors at Union Health and Family Welfare Centers level; and Community Health Care Providers, and Family Welfare Assistants at the Community Clinic/ Outreach level. Data may be obtained from the World Health Organization Country Office for Bangladesh upon reasonable request (Focal Point: Mr. Md Nuruzzaman- nuruzzamanm@who.int).

Competing interests

The authors declared that they do not have any competing interests.

Funding

This study was funded by WHO Country Office, Bangladesh and conducted by a group of researchers at BRAC James P Grant School of Public Health, BRAC University.

Authors' contributions

TJ conceived and designed the study. TJ and SNBKT carried out the data analyses and drafted the manuscript. MN, SA, VOC, and TZ thoroughly reviewed the manuscript and contributed substantially with necessary revision. TJ and SNBKT again reviewed the manuscript and prepared for final submission. All authors approved the final manuscript.

For peer review only

Acknowledgements

BRAC James P Grant School of Public Health, BRAC University acknowledges with gratitude to WHO Country Office, Bangladesh for its contribution in this research effort and MoHFW for supporting the research team throughout the study period. We are also thankful to the study participants who consented to participate in the study. We are indebted to Professor. Dr. Sayed Masud Ahmed, who served as the Advisor to the study project, and provided valuable technical inputs at different stages of the project. Finally, we would like to extend our sincere gratitude to the Field Supervisors and Data Collectors for their contribution throughout the qualitative and quantitative data collection.

Patient and Public Involvement statement: No patient involved

References

1. International Labor Organization. Global Extension of Social Security. Geneva; 2013.
2. World Health Organization. Global strategy on human resources for health: workforce 2030. Geneva; 2016.
3. WHO South East Asia Regional Office. Decade for health workforce strengthening in the South-East Asia Region 2015-2024, second review of progress; New Delhi; 2018 http://apps.searo.who.int/PDS_DOCS/B5439.pdf
4. Lerberghe WV, Conceic CC, Damme WV, and Ferrinho P. When staff is underpaid : dealing with the individual coping strategies of health personnel. 2002; 80 (01): p. 581–584.

For peer review only

https://www.ncbi.nlm.nih.gov/pmc/articles/PMC2567566/pdf/12163923.pdf.

5. Rowe AK, De Savigny D, Lanata CF, Victora CG. How can we achieve and maintain high-quality performance of health workers in low-resource settings?. *The Lancet*. 2005 Sep 17;366: 9490.p.1026-35. https://www.thelancet.com/journals/lancet/article/PIIS0140-6736(05)67028-6/fulltext?code=lancet-site.

6. World Health Organization. The world health report: working together for health. Geneva: World Health Organization; 2006.

7. Henderson LN, Tulloch J. Incentives for retaining and motivating health workers in Pacific and Asian countries. *Human resources for health*. 2008 Dec;6(1):18. doi: 10.1186/1478-4491-6-18.

8. Ahmed SM, Evans TG, Standing H, Mahmud S. Harnessing pluralism for better health in Bangladesh. *The Lancet*. 2013 Nov 23;382(9906):1746-55.doi: 10.1016/S0140-6736(13)62147-9.

9. Ahmed SM, Alam BB, Anwar A, Begum T, Haque R, AM Khan J and et al. Bangladesh Health System Review. Edited by A. Naheed and K. Hort. Dhaka: World Health Organization; 2015

10. Cometto G, Witter S. Tackling health workforce challenges to universal health coverage: setting targets and measuring progress. Bulletin of the World Health Organization. 2013;91:881-5. doi: 10.2471/BLT.13.118810.

11. Government of Bangladesh (2015) Bangladesh Health Workforce Strategy 2015. Dhaka.

12. Darkwa EK, Newman MS, Kawkab M, Chowdhury ME. A qualitative study of factors influencing retention of doctors and nurses at rural healthcare facilities in Bangladesh. *BMC health services research.* 2015 Dec;15(1):344. doi: 10.1186/s12913-015-1012-z.

13. Rawal LB, Joarder T, Islam SM, Uddin A, Ahmed SM. Developing effective policy strategies to retain health workers in rural Bangladesh: a policy analysis. *Human resources for health*. 2015 Dec;13(1):36. Available at: http://www.coe-uhc.org/index.php/publications/category/9-working-

For peer review only - http://bmjopen.bmj.com/site/about/guidelines.xhtml

paper-series.

14. deVaron Reynolds J, Costello T, Edwards MT. The study of workload in child protective and child welfare services. Time and Effort: Perspectives on Workload Roundtable. 2008 Dec 3. Available at: https://www.researchgate.net/profile/Robin_Perry3/publication/237430886_A_Critical_Appraisal_of_What_Child_Welfare_Workers_Do_Findings_From_a_Task_Analysis_Study_in_Florida/links/54662f200cf25b85d17f5b5b.pdf#page=4.

15. World Health Organization. Global strategy on human resources for health: workforce 2030. 2016. Available at: http://apps.who.int/iris/bitstream/10665/250368/1/9789241511131-eng.pdf?ua=1.

16. World Health Organization (2010) Workload Indicators of Staffing Need: User's Manual. Geneva: World Health Organization.

World Health Organization. Workload indicators of staffing need: User's manual. Geneva: World Health Organization. 2010.

17. Lopetegui M, Yen PY, Lai A, Jeffries J, Embi P, Payne P. Time motion studies in healthcare: what are we talking about?. *Journal of biomedical informatics*. 2014 Jun 1;49:292-9. doi: 10.1016/j.jbi.2014.02.017.

18. Government of Bangladesh. *Health Bulletin*. Dhaka. 2016a.

19. Ritchie J, Lewis J, Nicholls CM, Ormston R, editors. Qualitative research practice: A guide for social science students and researchers. sage; 2013 Nov 1.

20. Government of Bangladesh. Bangladesh Essential Health Service Package (ESP). Dhaka. 2016b.

21. Ahmed SM, Hossain MA, RajaChowdhury AM, Bhuiya AU. The health workforce crisis in Bangladesh: shortage, inappropriate skill-mix and inequitable distribution. Human resources for health. 2011 Dec;9(1):3. Available at: http://www.human-resources-health.com/content/9/1/3.

For peer review only - http://bmjopen.bmj.com/site/about/guidelines.xhtml

22. Greenglass ER, Burke RJ, Moore KA. Reactions to increased workload: Effects on professional efficacy of nurses. Applied psychology. 2003 Oct;52(4):580-97. doi: 10.1111/1464-0597.00152.

23. McQuide PA, Kolehmainen-Aitken RL, Forster N. Applying the workload indicators of staffing need (WISN) method in Namibia: challenges and implications for human resources for health policy. *Human resources for health*. 2013 Dec;11(1):64. doi: 10.1186/1478-4491-11-64.

24. Namaganda G, Oketcho V, Maniple E, Viadro C. Making the transition to workload-based staffing: using the Workload Indicators of Staffing Need method in Uganda. *Human resources for health*. 2015 Dec;13(1):89.. doi: 10.1186/s12960-015-0066-7.

25. Burmen B, Owuor N, Mitei P. An assessment of staffing needs at a HIV clinic in a Western Kenya using the WHO workload indicators of staffing need WISN, 2011. *Human resources for health*. 2017 Dec;15(1):9. doi: 10.1186/s12960-017-0186-3.

26. Ly A, Kouanda S, Ridde V. Nursing and midwife staffing needs in maternity wards in Burkina Faso referral hospitals. *Human resources for Health*. 2014 May;12(1):S8. doi: 10.1186/1478-4491-12-S1-S8.

27. Nayebi BA, Mohebbifar R, Azimian J, Rafiei S. Estimating nursing staff requirement in an emergency department of a general training hospital: Application of Workload Indicators of Staffing Need (WISN). *International Journal of Healthcare Management*. 2017 Oct 17:1-6. doi: 10.1080/20479700.2017.1390182.

28. Zaman S. Ladies without lamps: nurses in Bangladesh. Qualitative Health Research. 2009 Mar;19(3):366-74. doi: 10.1177/1049732309331876.

29. Ministry of Public Administration. 'Standard setup by the government for human resources in 10, 20, 31, 50, 100, 150, 200, 250, 500, 500 bed general hospitals at Union, Upazila, District, and Division levels, under Ministry of Health and Family Welfare'. Bangladesh. 2008.

For peer review only - http://bmjopen.bmj.com/site/about/guidelines.xhtml

Figure Legends

Figure 1: The ways WISN can help in human resource decision-making

Figure 2: Methods applied in each WISN step

Figure 3: Map of Jhenaidah and Moulvibazar Districts with study Upazilas identified

Figure 4: Approach of integration of ESP components in defining workload components of health service activity

For peer review only

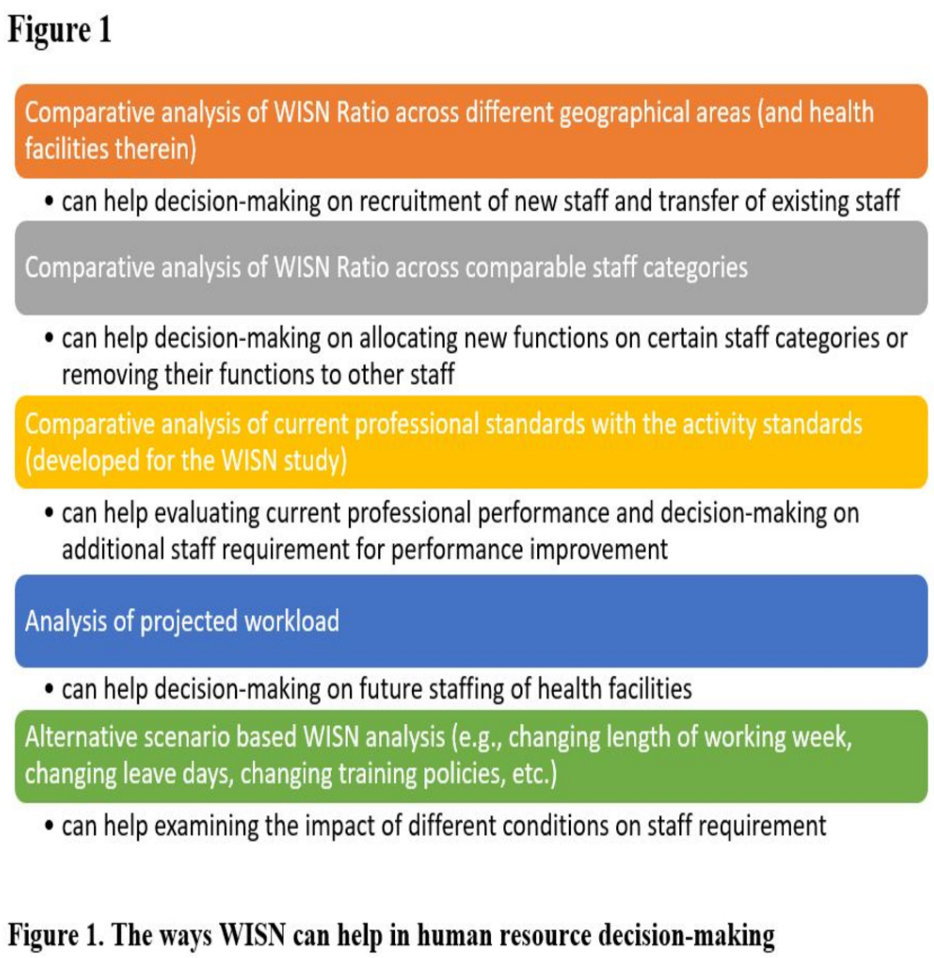

The ways WISN can help in human resource decision-making

Figure 2

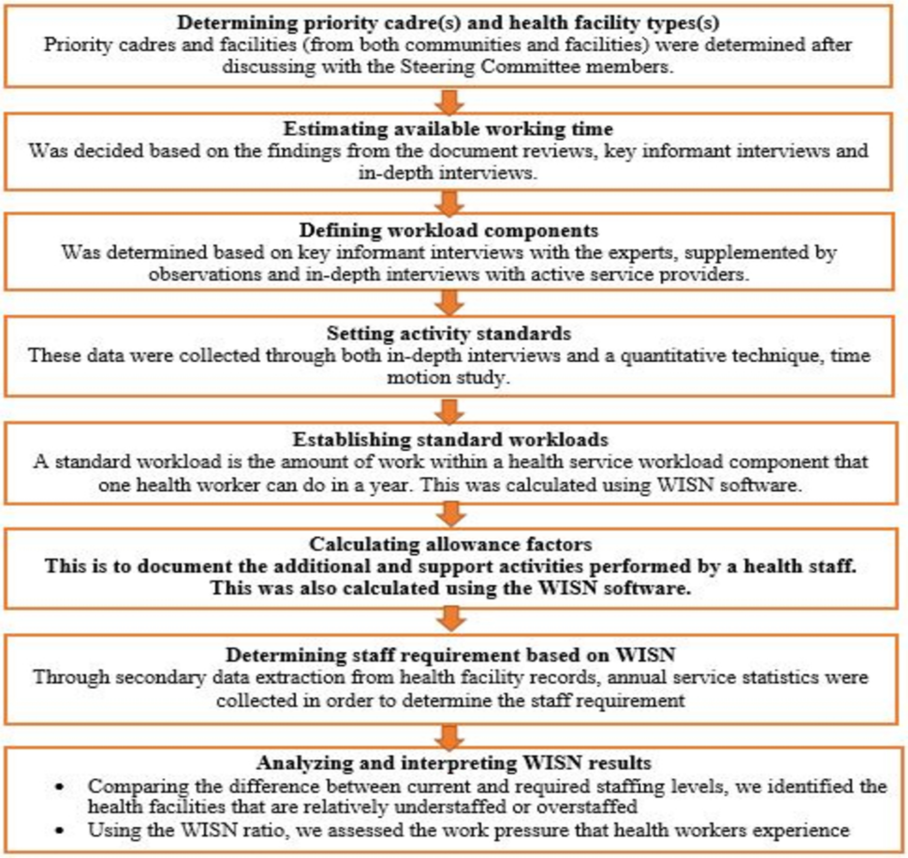

Figure 2 Methods applied in each WISN step

Methods applied in each WISN step

Figure 3

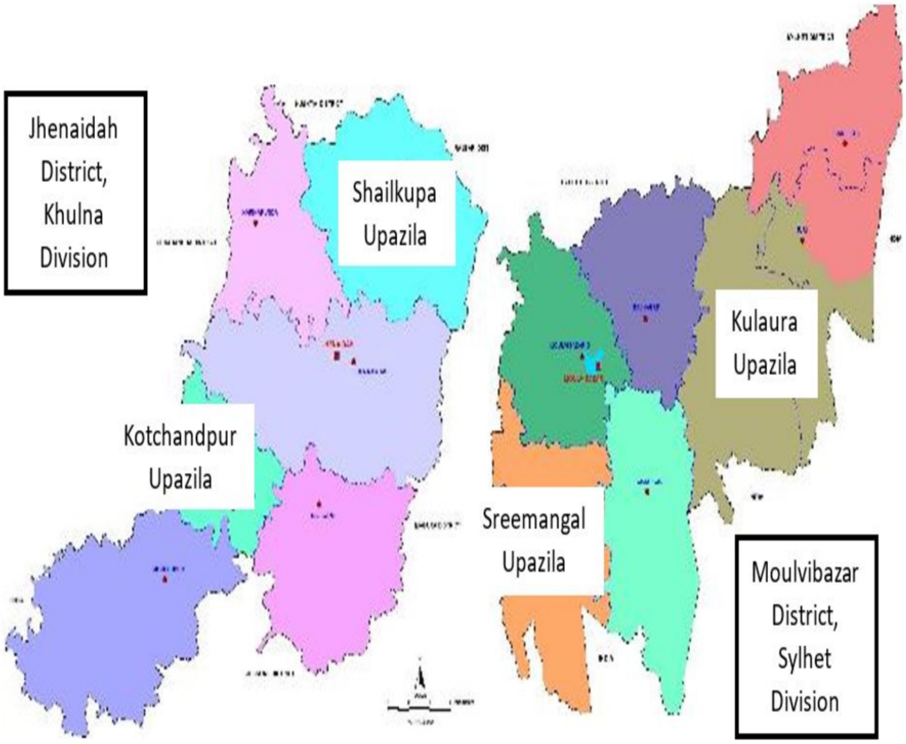

Figure 3 Map of Jhenaidah and Moulvibazar Districts with study Upazilas identified

Map of Jhenaidah and Moulvibazar Districts with study Upazilas identified

Figure 4

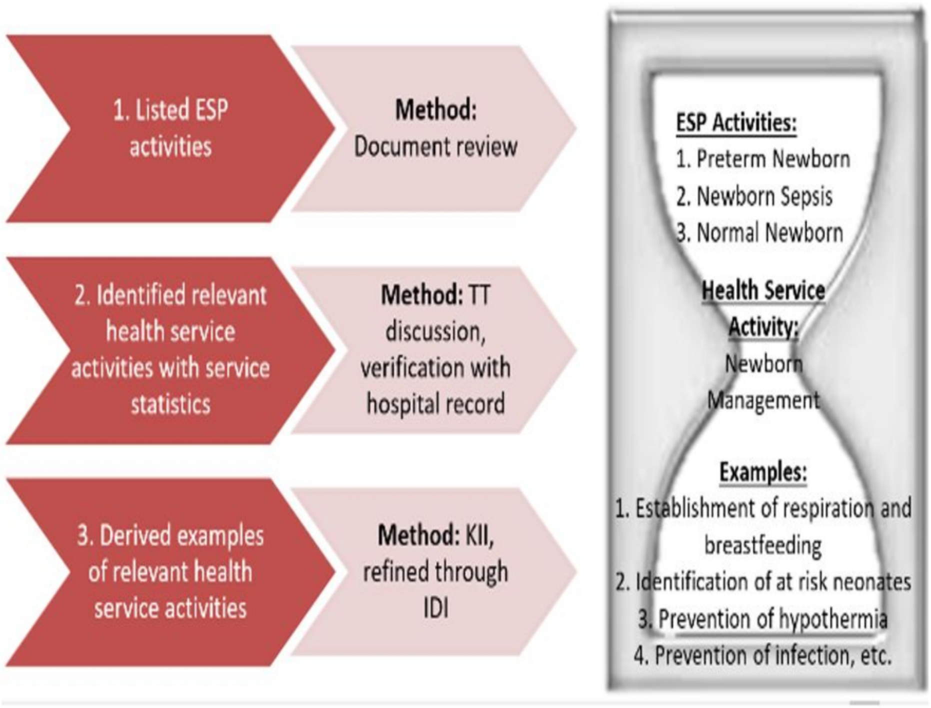

Figure 4 Approach of integration of ESP components in defining workload components of health service activity

Approach of integration of ESP components in defining workload components of health service activity

BMJ Open

An assessment of staffing needs for physicians and nurses at Upazila Health Complexes in Bangladesh using WHO workload indicators of staffing need (WISN) method

Journal:	*BMJ Open*
Manuscript ID	bmjopen-2019-035183.R2
Article Type:	Original research
Date Submitted by the Author:	09-Jan-2020
Complete List of Authors:	Joarder, Taufique; BRAC University, BRAC James P Grant School of Public Health; FHI 360, Bangladesh Office<br>Tune, Samiun; BRAC James P Grant School of Public Health, BRAC University, Public Health;<br>Nuruzzaman, Md; World Health Organization Bangladesh<br>Alam, Sabina; Government of Bangladesh Ministry of Health and Family Welfare, Health Services Division<br>Cruz, Valeria; World Health Organization, Country Office for Bangladesh<br>Zapata, Tomas; SEARO
**Primary Subject Heading**:	Health services research
Secondary Subject Heading:	Health policy, Global health, Nursing, Public health
Keywords:	Health policy < HEALTH SERVICES ADMINISTRATION & MANAGEMENT, PUBLIC HEALTH, HEALTH SERVICES ADMINISTRATION & MANAGEMENT, Human resource management < HEALTH SERVICES ADMINISTRATION & MANAGEMENT

SCHOLARONE™
Manuscripts

I, the Submitting Author has the right to grant and does grant on behalf of all authors of the Work (as defined in the below author licence), an exclusive licence and/or a non-exclusive licence for contributions from authors who are: i) UK Crown employees; ii) where BMJ has agreed a CC-BY licence shall apply, and/or iii) in accordance with the terms applicable for US Federal Government officers or employees acting as part of their official duties; on a worldwide, perpetual, irrevocable, royalty-free basis to BMJ Publishing Group Ltd ("BMJ") its licensees and where the relevant Journal is co-owned by BMJ to the co-owners of the Journal, to publish the Work in this journal and any other BMJ products and to exploit all rights, as set out in our licence.

The Submitting Author accepts and understands that any supply made under these terms is made by BMJ to the Submitting Author unless you are acting as an employee on behalf of your employer or a postgraduate student of an affiliated institution which is paying any applicable article publishing charge ("APC") for Open Access articles. Where the Submitting Author wishes to make the Work available on an Open Access basis (and intends to pay the relevant APC), the terms of reuse of such Open Access shall be governed by a Creative Commons licence – details of these licences and which Creative Commons licence will apply to this Work are set out in our licence referred to above.

Other than as permitted in any relevant BMJ Author's Self Archiving Policies, I confirm this Work has not been accepted for publication elsewhere, is not being considered for publication elsewhere and does not duplicate material already published. I confirm all authors consent to publication of this Work and authorise the granting of this licence.

For peer review only

**Title page

An assessment of staffing needs for physicians and nurses at Upazila Health Complexes in Bangladesh using WHO workload indicators of staffing need (WISN) method

Taufique Joarder[1, 2*], Samiun Nazrin Bente Kamal Tune[1], Md Nuruzzaman[3], Sabina Alam[4], Valeria de Oliveira Cruz[5], Tomas Zapata[5]

[1]BRAC James P Grant School of Public Health, BRAC University, Dhaka

[2]FHI360, Dhaka, Bangladesh

[3]World Health Organization Country Office for Bangladesh, Dhaka

[4]Health Services Division, Ministry of Health and Family Welfare, Government of Bangladesh

[5]World Health Organization South-East Asia Regional Office, New Delhi

Email addresses:

TJ: taufiquejoarder@gmail.com

SNBKT: samiun.tune@gmail.com

MN: nuruzzamanm@who.int

SA: alam6350@gmail.com

VOC: deoliveiracruzv@who.int

TZ: zapatat@who.int

*Corresponding author

For peer review only

Abstract

Objective This study aimed to assess the current workload and staffing need of Physicians and Nurses for delivering optimum health care services at the Upazila Health Complexes (UpHCs) in Bangladesh.

Design Mixed-methods, combining qualitative (e.g., document reviews, key informant interviews, in-depth interviews, observations) and quantitative methods (time-motion survey)

Setting Study was conducted in 24 health facilities of Bangladesh. However, UpHCs being the nucleus of primary health care in Bangladesh, this manuscript limits itself to reporting the findings from the providers at four UpHCs under this project.

Participants 18 Physicians and 51 Nurses, males and females.

Primary outcome measures Workload components were defined based on inputs from five experts, refined by nine service providers. Using WHO WISN software, standard workload, category allowance factor, individual allowance factor, total required number of staff, WISN Difference, and WISN Ratio were calculated.

Results: Physicians have Very High (WISN Ratio 0.43) and Nurse High (WISN Ratio 0.69) workload pressure. 50% of Nurses' time are occupied with support activities, instead of nursing care. There are different workloads among the same staff category in different health facilities. If only the vacant posts are filled, the workload is reduced. In fact, sanctioned number of Physicians and Nurses is more than actual need.

Conclusions: It is evident that high workload pressures prevail for Physicians and Nurses at the UpHCs. This reveals high demand for these health workforces in the respective sub-districts. WISN method can aid the policy-makers in optimizing utilization of existing human resources. Therefore, the government should adopt flexible health workforce planning and recruitment policy

For peer review only - http://bmjopen.bmj.com/site/about/guidelines.xhtml

to manage the patient load and disease burden. WISN should thus be incorporated as a planning tool for health managers. There should be a regular review of health workforce management decisions, and these should be amended based on periodic reviews.

Strengths and limitations

- Time-motion findings helped the experts suggest a more context sensitive activity standards.
- Using both qualitative and quantitative methods for primary data collection complemented each other for bringing data accuracy.
- Technical inputs from the WHO technical experts in WISN application in other countries, and officials of the Ministry of Health improved the data quality as they were directly involved in quality checks at the field level.
- One limitation was that some service statistics data, essential for establishing standard workloads, were unavailable.
- Due to lack of scope in WISN methodology, patient engagement was minimal.

Keywords

Human Resources for Health, Workforce Management, Workload Indicators of Staffing Need, Bangladesh, Upazila Health Complex, Health Systems

For peer review only - http://bmjopen.bmj.com/site/about/guidelines.xhtml

Background

Shortage of Human Resources for Heath (HRH) has been one of the major challenges faced by the health system and globally, more than 90 countries are haunted by this crisis. According to International Labor Organization (ILO), there are on average 34.5 health workers per 10,000 population and about one third of the world's population lack access to health care because of shortage of health workforce[1]. According to the Global strategy on human resources for health: workforce 2030, the estimated global shortage of skilled health workers will be around 18 million by 2030[2]. This problem has reached a critical stage in three (Bangladesh, Myanmar and Bhutan) South-East Asia Region countries with <23 health workers (doctors, nurses and midwives) per 10,000 population, limiting access to health services[3].

Improving health workers' performance and productivity is vital for better health service provision in the country. Poor performance of the health workers has been reported in the literature resulting from too few staff, or staff not providing care according to standards[4–6]. The extent of the shortage is reflected in health worker density rates and workforce vacancy rates, and its impact in health system performance indicators. Factors that contribute to poor performance of health workers include limited employment opportunities and low salaries; poor working conditions, weak support and supervision, and limited opportunities for professional development[7].

Bangladesh's health workforce scenario is characterized by "shortage, inappropriate skill mix and inequitable distribution"[8,9]. Equitable access to skilled and motivated health worker in a functional health system is essential for achieving Universal Health Coverage (UHC) and the Sustainable Development Goals[10]. In 2015, Government of Bangladesh (GoB) approved the Bangladesh

For peer review only - http://bmjopen.bmj.com/site/about/guidelines.xhtml

Health Workforce Strategy which affirms government's vision of equitable availability of skilled, motivated and responsive health workforce in adequate numbers across the country[11]. However, there is lack of comprehensive, nationally representative data on HRH workload and optimum staff need in health care facilities in Bangladesh. A small scale qualitative study found overwhelming workload as one of the critical components that hinders retention of doctors and nurses at rural healthcare facilities in Bangladesh[12]. Another policy analysis on retention of HRH (physicians and nurses) also found that deficiency of adequate workforce and consequent high workload acted as a deterrent against rural retention[13].

Workload management is very important for any country or institution to deliver quality services, retain staffs and reduce turnover[14]. Even the seminal document on HRH, "Global strategy on human resources for health: Workforce 2030", emphasized on developing country level workforce strategies, drawing on workload analysis studies[2]. Such studies can provide detailed insight into the current state of workload in a system, coping strategies of the staff for regular extra work pressure, causes behind the excessive workloads, and ways to deal with it. This study aimed to fill-in this knowledge gap with respect to workload and optimum staff need for physicians and nurses at the Upazila or Sub-district level (i.e., at Upazila Health Complex [UpHC]). It is expected that this workload analysis will contribute in improving performance, ensuring quality of services, and facilitating uninterrupted service delivery through efficient management of staff.

Workload Indicator of Staffing Need (WISN) overview

WHO developed the Workload Indicator of Staffing Need (WISN) method in 1998, which was later updated based on learning from implementation in different countries. This method is simple,

For peer review only - http://bmjopen.bmj.com/site/about/guidelines.xhtml

useful and time-saving, which was borrowed from the industrial sector for use in the health sector by Peter Shipp in 1984. The result is expressed in terms of differences and ratios, the former indicating worker shortage or surplus, and the latter workload pressure experienced by the staff[15].

WISN results help in human resource decision-making in several ways (Figure 1). For example, recruitment and transfer of HRH can be based on geographic comparison of WISN ratios, staffing of health facilities can be informed by WISN-based workload projection, etc.

Methods

Study design

We followed the updated WISN manual[15], but contextualized it for Bangladeshi setting. The WISN steps have been summarized in Figure 2. The research project was developed based on close collaboration among and mutual insights from three types of committees:

1. **Steering Committee (SC):** The SC was consisting 13 members, established by the Ministry of Health and Family Welfare (MOHFW) with membership from senior government officials (seven): WHO official (one); professional organization of the physicians, Bangladesh Medical Association (one); and relevant academia (four) such as BRAC University (two persons), Bangladesh University of Health Sciences, and Center for Medical Education. All seven senior government officials were directly involved in decision making regarding daily management of the health workforce in their respective departments. WHO officer was there to respond to WISN related technical issues and application. All four academicians were part of the committee because they had expertise in their respective areas (i.e. education, policy making, curriculum development and performance assessment) of the health workforce. They were also

For peer review only - http://bmjopen.bmj.com/site/about/guidelines.xhtml

well known in the community of scientific writing and academic teaching. The role of the SC was to guide and endorse the overall study based on the WISN strategy and its implementation.

2. **Technical Taskforce (TT):** TT was responsible for guiding the implementation of the WISN process. Researchers from implementing research institution (the school of public health of a Bangladeshi university, BRAC University); and experts from WHO Bangladesh Country Office; an international non-governmental organization (NGO), Save the Children; and another university, Bangladesh University of Health Sciences served in the Taskforce.
3. **Expert Working Groups (EWG):** There were multiple EWGs, one for each of the following professional groups: General Physicians (Medical Officer [MO], Emergency Medical Officer [EMO], Residential Medical Officer [RMO]), and Nursing Staff (Senior Staff Nurse, Nursing Supervisor). The respective EWG defined the workload components and set activity standard for the specific staff category.

The qualitative part of this research involved document reviews, key informant interviews (KII) with policy level persons related to HRH issues in Bangladesh (mostly from among SC and EWG members), in-depth interviews (IDI) with individual service providers (e.g., physicians, nurses, etc. working in UpHCs under this study), and observations. The quantitative component involved time-motion survey which is a work measurement technique for recording the times and rates of working for the elements of a specific job though observing a subject continuously or in a certain period of time[16]. Time motion data served as a guide to determine the activity standard for WISN analyses.

For peer review only - http://bmjopen.bmj.com/site/about/guidelines.xhtml

Study duration, setting and population

The research continued from July to November 2017 and was carried out in two pre-selected Districts of Bangladesh (Figure 3):

- Jhenaidah, located in south-western part of Bangladesh, under Khulna Division
- Moulvibazar, located in north-eastern part of Bangladesh, under Sylhet Division

The selection of the districts was made by the Human Resources Unit, Ministry of Health and Family Welfare **in collaboration with the development partners (Save the Children and WHO Bangladesh) in a workshop nationally organized in November 2016. Later the selection of the UpHCs was made in consultation with the SC, taking into consideration some performance indicators such as** number of beds, number of total deliveries, number of live births, bed occupancy rates as well as patient load including the number of outpatient visits. **From each district, two highest performing UpHCs were included in the study**[17]. Thus, Shailkupa and Kotchandpur UpHCs were selected from Jhenaidah District and Kulaura and Sreemangal from the Moulvibazar. Because future applications need to be based on optimum quality standards, the highest performing UpHCs were selected to serve as a model for other health facilities. From each of these four UpHCs, physicians (Medical Officers and Residential Medical Officers) and nurses (Senior Staff Nurse and Nursing Supervisor) were included for workload analysis. A total of 24 health facilities from the two districts were studied. This included two district hospitals, four UpHCs, two Maternal and Child Welfare Centers, eight Union Sub Centers, eight Union Health and Family Welfare Centers, and eight Community Clinics. This manuscript reported findings from all the four UpHCs under this study, as UpHCs are the nucleus of the primary health care delivery in Bangladesh, serving the rural population.

For peer review only - http://bmjopen.bmj.com/site/about/guidelines.xhtml

Sampling strategy

Qualitative part

Documents for review were selected based on the suggestions from the experts (members of SC, TT, and EWG), supplemented by reference tracking of government reports and published literature on HRH of Bangladesh. Key Informants were selected on the principles of purposive sampling[18], supplemented by snowball sampling (i.e., based on the reference or suggestion from the key informants). IDI respondents were selected through purposive sampling, based on the respondent's seniority and designation (e.g., Residential Medical Officer, Nursing Supervisors, etc.). These respondents were practicing individuals and had more than 10 years of experience and played a supervisory role in their respective health facilities.

Quantitative part

For time-motion study, time sampling was done for each consenting staff available during the data collection period. Field Data Collectors (FDCs) observed each staff twice for 45-minutes duration, once during the first half of their service duration and again during the second half. This was done to minimize the bias in the time data due to the patient load (assuming higher patient load in the first half and lower in the second).

For peer review only - http://bmjopen.bmj.com/site/about/guidelines.xhtml

Tool development, pretest, training of data collectors, agreement test

For qualitative data collection, semi-structured guidelines, including that for document reviews, KIIs, IDIs, and observation, were developed. For the time-motion study, structured observation tool was designed. The structured observation tools contained three sections:

1. Background information of observation setting and the person under observation
2. Time-motion data sheet (containing three columns: type of activity, time spent in minutes, and remarks)
3. Example of the activities (health service activities, support activities, and additional activities)

Health service activities, according to WISN manual, are performed by all members of the staff category and regular service statistics are available for them for example, obstetrical service, emergency service, outpatient service, etc. Support activities are also performed by all members of the staff category, but regular service statistics are not available for them for example, record keeping and reporting, attending meetings, instrument sterilization, etc. Additional activities are performed by only certain members of the staff category (e.g., the supervisor or a senior member), and regular service statistics are not available for them for example, duty roster preparation, preparing staff evaluation reports, supervision of cleanliness, etc.[15].

The examples of health service activities were primarily drawn from the list of the activities mentioned in the Essential Services Package (ESP)[19] for respective health facility type. Since service statistics were not available according the ESP activity list, it was adjusted for the local context with inputs from the respondents (through KII, followed by IDI), in alignment with the

For peer review only - http://bmjopen.bmj.com/site/about/guidelines.xhtml

availability of service statistics. An ‘hourglass’ approach was adopted for defining the workload components based on the ESP (Figure 4). Tools were pre-tested in a UpHC near Dhaka, before applying for actual data collection. Qualitative tools were also pre-tested through mock IDIs and KIIs. The pre-testing exercise was followed by the training of the Field Supervisors (FSs) and Field Data Collectors (FDCs).

Data collection and quality control

At first, FSs were sent to respective districts to orient the personnel on the project, seek support, and assess the availability of the service statistics. The FSs spent one week in each district and conducted qualitative observation of the service provision at the UpHCs, to gain a firsthand understanding of the context.

In the second step, we conducted five KIIs to define workload components, in light of the ESP document’s standard of services by facility level[19]. The workload components were further refined based on IDIs with a total of eight physicians and eight nurses.

In the third step, the FDCs, under the supervision of FSs, conducted time-motion study, using a mobile device (SurveyCTO software). During the time FDCs were collecting time-motion data, the FS, in addition to supervising the FDCs, conducted additional IDIs and collected data on available working time; time required for health service, support, and additional activities; and service statistics.

For peer review only - http://bmjopen.bmj.com/site/about/guidelines.xhtml

The next step was data validation and set activity standards. Primary data validation was done through phone calls made to the services providers, and health facility statisticians. Secondly, these were shared with the SC and TT members. Finally, interviews were conducted with the EWG members to finalize the activity standards (Table 1).

Table 1 Number of interviewees representing EWG of staff categories

EWG representing staff category	Number
Physicians: MO, EMO, RMO	2
Nurses: Nursing Supervisor, Senior Staff Nurse	7
Total	9

We presented the time-motion findings to the EWG members and requested them to be as realistic as possible in suggesting activity standards. We also requested them to account for the variability of patient load due to factors such as seasonality, timing of day, facility catchment population, etc. Finally, through multiple meetings, debates and deliberations, the activity standard was finalized, taking into account the information from the IDIs and KIIs, and inputs from the EWG members (Table 2).

Table 2 Service standard for Physicians and Nurses in UpHCs, Bangladesh, 2017

Activities	Service standard		Unit
	Physician	Nurse	

For peer review only - http://bmjopen.bmj.com/site/about/guidelines.xhtml

Obstetrical Service (Caesarean Section)[a]	90	90	min/patient
Obstetrical Service (Normal Delivery)	60	120	min/patient
Newborn Management	15	15	min/inpatient day
Emergency Service	15	Not applicable	min/patient
IMCI/ Nutritional Service	15	15	min/patient
OPD Service (including NCD management)	10	Not applicable	min/patient
First ANC	20	20	min/patient
Follow-up ANC	10	10	min/patient
PNC	15	15	min/patient
Indoor Services (Round, including minor bedside procedures)[b]	25.65	34	min/inpatient day
Bedside patient care	Not applicable	17	min/inpatient day
Patient admission and discharge	Not applicable	20	min/inpatient day
Death certification and associated arrangements	20	30	min/patient

Note: [a]For Nurses: Assist Obstetrical Service (Caesarean Section); [b]For Nurses: Indoor Services (Round with Physician)

IMCI: Integrated Management of Childhood Illness; OPD: Out Patient Department; NCD: Non-communicable Diseases; ANC: Antenatal Care; PNC: Postnatal Care.

For peer review only - http://bmjopen.bmj.com/site/about/guidelines.xhtml

In order to ensure the quality of data, the Principal Expert (lead author of this article), Co-experts (two co-experts – one was leading data collection and the other was leading data quality check and reporting), WHO team consisting of national and international technical experts, and officials from the Human Resource Branch of Ministry of Health and Family Welfare (MoHFW) conducted field visits to each study District and the health facilities therein. During the time-motion data collection period, the Co-experts monitored the data and their geographic location in real-time. They also regularly checked the consistency of the data. Our field based data collection team saved contact information of all the respondents; so, in case of any confusion or need for clarification, the Co-experts called the respondents over phone.

Data management and analysis

The first analytic step was to estimate available working time of the staffs. This is the time a health worker has available in one year to do his or her work, taking into account authorized and unauthorized absences[15]. For all categories of staff, a uniform number of weeks per year (52 weeks), working days in one week (six days), possible working days in one year (52 * 6 = 312 days) were estimated. Next, absent days, such as public holidays (20 days), earned leave (average for each staff category, based on Health Management Information System data), and casual leave (20 days) were deducted to obtain the annual working time in days. Multiplying this with daily working hours (six hours per day), we obtained annual working time in hours.

For peer review only - http://bmjopen.bmj.com/site/about/guidelines.xhtml

Workload components were defined through the inputs from the key informants; activity standards were also set through the interviews with the EWG members. An activity standard is the time necessary for a well-trained, skilled and motivated worker to perform an activity to professional standards in the local circumstances[15]. Both service standards (for health service activities), category allowance standards (for support activities), and individual allowance standards (for additional activities) were determined in the same way.

The next analytic step was to establish standard workload, which was done by dividing the annual working time by unit time of health service activities. A standard workload is the amount of work within a health services workload component that one health service provider can do in a year hypothetically[15]. Then category allowance factor and individual allowance factors were calculated using the following formula, respectively:

Category allowance factor = 1 / {1 – (Total category allowance standard / 100)}

Individual allowance factor = Total individual allowance standard / Available working time in hours

Next, exact number of required staff was calculated by the following formula:

Total required number of staff = (Staff needed for health service activity * Category allowance factor) + Individual allowance factor

The fractional results were rounded up or down, following the guideline provided in the WISN manual[15]:

- 1.0 – 1.1 is rounded down to 1 and >1.1 – 1.9 is rounded up to 2
- 2.0 – 2.2 is rounded down to 2 and >2.2 – 2.9 is rounded up to 3
- 3.0 – 3.3 is rounded down to 3 and >3.3 – 3.9 is rounded up to 4

For peer review only - http://bmjopen.bmj.com/site/about/guidelines.xhtml

- 4.0 – 4.4 is rounded down to 4 and >4.4 – 4.9 is rounded up to 5
- 5.0 – 5.5 is rounded down to 5 and >5.5 – 5.9 is rounded up to 6

Finally, based on the existing number of staff in the respective health facilities, we calculated both the difference (current number of staff – required number of staff by WISN), and the ratio (current number of staff / required number of staff by WISN). The WISN difference indicates whether the health facilities are relatively understaffed (i.e., when the WISN difference is negative), overstaffed (i.e., when the WISN difference is positive), or balanced (i.e., when the WISN difference is zero). The WISN Ratio indicates whether the staffs are experiencing high workload (i.e., when the WISN Ratio is lower than one), low workload (i.e., when the WISN Ratio is higher than one), or normal workload (i.e., when the WISN Ratio is equal to one). For this calculation, we used the de-facto number of current staff, i.e., the number of staff that we actually found working in the health facilities during our data collection period; not the number shown in the office records or statistics.

For peer review only

Ethical considerations

Ethical approval for this study was obtained from the Ethical Review Committee (ERC) of BRAC James P Grant School of Public Health, BRAC University. All ethical principles were strictly adhered to. We obtained a support letter from the Ministry of Health and Family Welfare and also obtained written informed consent form from each person we engaged in data collection. Identity of respondents was kept confidential.

For peer review only - http://bmjopen.bmj.com/site/about/guidelines.xhtml

Results

General WISN findings across levels

For descriptive purposes, we have categorized the workload pressure as Extremely High (WISN Ratio between 0.10 and 0.29), Very High (WISN Ratio between 0.30 and 0.49), High (WISN Ratio between 0.50 and 0.69), Moderately High (WISN Ratio between 0.70 and 0.89), Normal (WISN Ratio between 0.90 and 1.19), and Low (WISN Ratio greater than or equal to 1.20). Based on this categorization, at an aggregate level (i.e., considering the average required number and WISN Ratio across the same types of health facilities), physicians are found to have a Very High (WISN Ratio 0.43) and nurses High (WISN Ratio 0.69) workload pressure. To cope with the workload, on an average 11 physicians (on average 4.50 were available during data collection) and 19 nurses (On average 12.75 were available during data collection) are needed in each UpHC (Table 3). This means, there was an average deficit of six members in each staff categories.

Table 3 Analysis of WISN results at aggregate level (average required number and WISN Ratio across same types of health facilities)

Staff category	Required staff to cope with the demand	Average number of existing staff	Deficit of staff	Average WISN Ratio	Workload pressure
Physician	10.59	4.50	6.09	0.43	Very high
Nurse	18.86	12.75	6.11	0.69	High

For peer review only - http://bmjopen.bmj.com/site/about/guidelines.xhtml

Tabulating the total percentage of time spent on all support activities (i.e., category allowance standards) by different staff categories, we found that, 50% of nurses' time are occupied with support activities (Table 4).

Table 4 Comparison of Support Activities across staff categories

Staff category	Total % of support activities
Physician	24%
Nurse	50%

WISN results disaggregated by UpHCs

The required number of staff ranges from eight to 12 among Physicians, and 16 to 23 among Nurses. Highest shortage is observed in Nurses of Sreemangal UpHC (-8.46), followed by Physicians of Kulaura UpHC (-8.28). Workload pressure is the highest among Physicians of Kotchandpur UpHC (WISN Ratio 0.28) and lowest among Nurses of Shailkupa (WISN Ratio 0.87) (Table 5).

Table 5 Analysis of WISN results of Upazila level health staff

Health facility	Current number of staff	Required number, based on WISN	Shortage or excess	WISN Ratio	Workload pressure
**Staff category: Physician**					

For peer review only - http://bmjopen.bmj.com/site/about/guidelines.xhtml

Shailkupa UpHC	4	8.14	-4.14	0.49	Very High
Kotchandpur UpHC	3	10.71	-7.71	0.28	Extremely High
Kulaura UpHC	4	12.28	-8.28	0.33	Very High
Sreemangal UpHC	7	11.23	-4.23	0.62	High
**Staff category: Nurse**					
Shailkupa UpHC	14	16.08	-2.08	0.87	Moderately High
Kotchandpur UpHC	15	22.80	-7.8	0.66	High
Kulaura UpHC	10	16.08	-6.08	0.62	High
Sreemangal UpHC	12	20.46	-8.46	0.59	High

For peer review only

Change of workload if vacancies are filled

If the vacant posts are filled, understandably, the workload is reduced. In most of the cases, sanctioned number of Physicians and Nurses is more than what is actually needed to tackle the workload. However, only filling up the vacant posts are not enough in case of some of the staff categories, such as the Nurses at Kotchandpur and Physicians at Sreemangal (Table 6).

For peer review only - http://bmjopen.bmj.com/site/about/guidelines.xhtml

Table 6 Change of workload if vacancies in Physician and Nursing posts are filled

Health facility	Staff category	Current number of staff	Required number, based on WISN	WISN Ratio	Sanctioned number of staff	WISN Ratio as per sanctioned number of staff
Shailkupa UpHC	Physician	4	8.14	0.49	10	1.25
	Nurse	14	16.08	0.87	21	1.31
Kotchandpur UpHC	Physician	3	10.71	0.28	20	1.82
	Nurse	15	22.80	0.66	20	0.87
Kulaura UpHC	Physician	4	12.28	0.33	20	1.67
	Nurse	10	16.08	0.62	26	1.62
Sreemangal UpHC	Physician	7	11.23	0.62	10	0.91
	Nurse	12	20.46	0.59	22	1.10

Discussion and recommendations

Discussion

Findings from this WISN study clearly indicates that the public-sector healthcare providers in Bangladesh are suffering from a very high workload pressure. Nurses are predominantly occupied with support activities rather than actual nursing care. There is unequal workload across UpHCs, indicating potential for workforce re-distribution. The unequal workload mainly stems from

For peer review only

For peer review only - http://bmjopen.bmj.com/site/about/guidelines.xhtml

differing patient load, due to geographic location, number of catchment population, and epidemiological characteristics, at different UpHCs. Inappropriate number of sanctioned posts indicate the necessity of WISN-based workforce planning.

High workload pressure may arise from absolute or relative shortage of health workforce. Absolute shortage appears when there is inadequate production of a particular staff category while relative shortage appears when health workforce is not distributed evenly between the urban and rural areas throughout the country for various reasons. For example, absolute shortage in HRH production is revealed by the fact that there are only 4.90 registered physicians and 2.90 registered nurses per 10,000 populations[17], rendering the country to be one of the 57 critical workforce shortage countries in the world[6]. On top of this absolute shortage, Bangladesh also suffers from relative shortage, as evidenced from the fact that physician to population ratio in urban areas is 1:1,500, but in rural areas it is 1:15,000[20]. Workload pressure has some serious consequences as well, namely, fatigue and burnout of service providers, lack of motivation, and compromised quality of care[21]. High workload is, however, not unique to Bangladesh. WISN studies in Low- and Middle-Income Countries (LMICs) like Namibia[22], Uganda[23], Kenya[24], Burkina Faso[25], and Iran[26] also identified high workload pressure among their HRH.

It is expected that the nurses would spend most of their service times beside the patients, providing nursing care. Unfortunately, this is not the case in Bangladesh as well as in some other comparable settings. A qualitative study in Bangladesh showed, nurses' maximum time is spent on administrative and paperwork tasks[27]. Excessive support activities of nurses is reported in studies conducted in Iran[26], and Uganda[23] as well. A recent WISN study conducted in Iran showed, nurses

For peer review only - http://bmjopen.bmj.com/site/about/guidelines.xhtml

are overburdened; and support activities account for 31% of their workload[26]. Nurses' excessive engagement in paperwork or other support activities may result from deficient human resource planning and management.

Despite the fact that most of the staff are already overworked, staffs in some health facilities may be more so, compared to a neighboring one. Presence of different number of staffs causes fluctuation in the amount of workload at different health facilities. In places where workload of a staff category is 'Extremely High', some supports from nearby health facilities with lower workload should be sought. Or, in places where workload of a staff category is 'Normal' or 'Low', some support may be transferred to health facilities with higher workload. For example, in Sreemangal UpHC, there are seven physicians, with a High workload pressure. However, workload pressure in nearby Kulaura UpHC is Very High, with only four physicians (Table 5). At least one physician from Sreemangal can be reallocated to Kulaura to tackle the high workload. Similar action may be taken regarding the Nurses by transferring some from Shailkupa (Moderately High workload) to Kotchandpur (High workload). This is just an example how WISN can help in decision-making regarding allocation of human resources. Similar situation was identified in Namibia, where researchers suggested redistribution of health workers from one area to the other[22].

We found that many posts remained vacant in different health facilities. Some staffs were not present at their service locations for various reasons, such as training, deputation to another health facility, etc. Even if the existing posts are filled-up, a large portion of the workload would be curbed. For example, according to the Standard Setup document of the Ministry of Public

For peer review only - http://bmjopen.bmj.com/site/about/guidelines.xhtml

Administration, 18 physician posts (10 Junior Consultants, one Residential Medical Officer, seven Assistant Surgeons) have been proposed for a 50 bed hospital[28]. We have found 4.5 Physicians on an average in each UpHC. The average required number is 11 (Table 3). Our proposition is that, even if it is not possible to reach the ideal workforce setup for a health facility, filling-up at least the vacant positions, and ensuring regular presence of all staffs would reduce the workload. Supportive supervision and monitoring of the staff is essential to ensure the presence of posted staff. Researchers in Namibia came up with the similar finding and proposed a similar solution for the problem[22]. WISN was used over standard staffing schedule in HIV Clinics in Kenya as well to resolve a similar crisis[24].

Recommendations

Based on the findings and its in-depth analysis, we propose few short-term and long-term recommendations. The short-term recommendations require administrative or management decisions, relatively easier to implement and tackle the immediate crisis. On the other hand, the long-term recommendations demand radical policy amendments following careful examination.

Short-term recommendations include: reallocation of staff from low workload areas to high workload areas, fill-up existing vacant positions and strengthen supervision and monitoring. Nurses are the most needed staff, the most overloaded, and are short in supply. On top of all these, they are burdened with support activities. If some of their support and additional activities can be shifted to other staff, nurses can devote their time better in nursing care.

For peer review only - http://bmjopen.bmj.com/site/about/guidelines.xhtml

The study yields some long-term recommendations as well, for the policy-makers. For example, in order to increase the availability of workforce, especially Nurses, and decrease their workload, their number needs to increase. Hence, long term policy response is needed to increase the intake of nursing students, train them with quality education, and deploy them in larger numbers in a secure and gender-friendly work environment. In the same vein, incentives should be given to increase the number of nurses in both public and private sector educational institutions. Regulations should be developed and implemented so that medical colleges can be established only when a nursing school is established alongside. Otherwise, the skill-mix imbalance between physicians and nurses would jeopardize the quality of care. Quality and quantity of physicians should also increase.

Secondly, since the Nurses are found to be predominantly engaged in support activities at the expense of actual patient care, a separate staff category for administrative/ support activities is greatly warranted. This will free up the valuable yet scarce clinical time of the service providers.

Thirdly, instead of the existing approach of deploying a fixed number of workforce at all health facilities, a flexible recruitment and HRH planning is needed, based on patient load and disease burden. This can be supported by determination of optimal requirements of HRH under given limited resources or constraints in those health facilities[29–32] by using the WHO methodology on workload indicators of staffing need. It is important to recognize that, decisions in health sector are very much contingent on the local context, especially the patient load, demographic drivers (e.g., age structure of the population, gender ratio, etc.), and epidemiologic profile. Therefore, the government should adopt flexible health workforce planning and recruitment policy in place to keep up with the local patient load and disease burden. The culture of bottom-up decision-making should be adopted eventually.

For peer review only - http://bmjopen.bmj.com/site/about/guidelines.xhtml

Strengths and limitations

This study had a number of strengths. First, we conducted time-motion study, which helped the research team gain a better understanding of the service context of the staffs. Secondly, when the key informants or experts suggested an unrealistic activity standard, we presented them the time-motion findings and helped them suggest a more context sensitive standards. Thirdly, the research team used both qualitative and quantitative methods for primary data collection, which complement each other for bringing data accuracy. Fourthly, WHO technical officers, who had expertise in WISN application in other countries, and the Ministry of Health officials were directly involved in the field level data quality checks.

However, despite careful planning and painstaking implementation of the research, we faced some challenges during different stages of the WISN process. First, some service statistics data, which were essential for establishing standard workloads, were not readily available due to poor record keeping systems at some health facilities. Secondly, the research did not take into account the patients' opinion or stakeholders' stance. Notwithstanding the fact that these perspectives are gaining momentum in health workforce decision-making, we could not take advantage of them in the interest of adhering to the highly structured nature of the WISN methodology. Thirdly, the official number of existing staffs often did not match with the number of staffs we observed providing services.

Conclusions

For peer review only - http://bmjopen.bmj.com/site/about/guidelines.xhtml

Human resource management is a big challenge, especially in a resource-poor setting like Bangladesh. With a vision of becoming a middle-income country by 2021, Bangladesh needs to strive for optimizing its existing resources, including human resources. This type of study can aid the decision-making in this direction, using the WISN as a planning tool for the managers. Implementation research is needed regarding how this workload-based staffing decisions can be integrated into the health systems in the most effective way. We expect that, these types of studies would pave the way for evidence-based HRH decision-making in the context of health system of Bangladesh.

For peer review only

List of abbreviations

ANC: Antenatal Care;

EMO: Emergency Medical Officer;

ERC: Ethical Review Committee;

ESP: Essential Services Package;

EWG: Expert Working Groups;

FDCs: Field Data Collectors;

FSs: Field Supervisors;

GoB: Government of Bangladesh;

HRH: Human Resources for Heath;

IDI: In-depth Interviews;

IMCI: Integrated Management of Childhood Illness;

KII: Key Informant Interviews;

LMICs: Low- and Middle-Income Countries;

For peer review only - http://bmjopen.bmj.com/site/about/guidelines.xhtml

MO: Medical Officer;

MoHFW: Ministry of Health and Family Welfare;

NCD: Non-communicable Diseases;

OPD: Out Patient Department;

PNC: Postnatal Care;

RMO: Residential Medical Officer;

SC: Steering Committee;

TT: Technical Taskforce;

UHC: Universal Health Coverage;

UpHCs: Upazila Health Complexes;

WISN: Workload Indicators of Staffing Need

Declarations

Consent for publication

During the data collection process, while the ethical consents were obtained from the respondents, they were informed that their data might be used for publication in future. They were also informed that their identity will remain anonymous. Institutional consents for publication were obtained as well.

Availability of data and materials

All research data have been submitted to the World Health Organization Country Office for Bangladesh, as per the agreement with the research organization, BRAC James P Grant School of Public Health, BRAC University. This manuscript only used the data pertaining to the Physicians

For peer review only - http://bmjopen.bmj.com/site/about/guidelines.xhtml

and Nurses working at the two study Upazila (sub-district) Health Complexes. Unpublished data include: Consultants, General Physicians, and Nurses at the District Hospital level; Physicians, and Family Welfare Visitors at Maternal and Child Welfare Center level; Sub-Assistant Community Medical Officers at the Upazila Health Complex level; Sub-Assistant Community Medical Officers at Union Sub-Centers level, Sub-Assistant Community Medical Officers, and Family Welfare Visitors at Union Health and Family Welfare Centers level; and Community Health Care Providers, and Family Welfare Assistants at the Community Clinic/ Outreach level. Data may be obtained from the World Health Organization Country Office for Bangladesh upon reasonable request (Focal Point: Mr. Md Nuruzzaman- nuruzzamanm@who.int).

Competing interests

The authors declared that they do not have any competing interests.

Funding

This study was funded by WHO Country Office, Bangladesh and conducted by a group of researchers at BRAC James P Grant School of Public Health, BRAC University.

Authors' contributions

TJ conceived and designed the study. TJ and SNBKT carried out the data analyses and drafted the manuscript. MN, SA, VOC, and TZ thoroughly reviewed the manuscript and contributed substantially with necessary revision. TJ and SNBKT again reviewed the manuscript and prepared for final submission. All authors approved the final manuscript.

For peer review only - http://bmjopen.bmj.com/site/about/guidelines.xhtml

Acknowledgements

BRAC James P Grant School of Public Health, BRAC University acknowledges with gratitude to WHO Country Office, Bangladesh for its contribution in this research effort and MoHFW for supporting the research team throughout the study period. We are also thankful to the study participants who consented to participate in the study. We are indebted to Professor. Dr. Sayed Masud Ahmed, who served as the Advisor to the study project, and provided valuable technical inputs at different stages of the project. Finally, we would like to extend our sincere gratitude to the Field Supervisors and Data Collectors for their contribution throughout the qualitative and quantitative data collection.

Patient and Public Involvement statement: No patient involved

Reference

1 International Labor Organization. Global Extension of Social Security. Geneva: 2013.

2 World Health Organization. *Global Strategy on Human Resources for Health: Workforce 2030*. Geneva: : World Health Organization 2016. http://apps.who.int/iris/bitstream/10665/250368/1/9789241511131-eng.pdf?ua=1

3 WHO South East Asia Regional Office. *Decade for health workforce strengthening in the South-East Asia Region 2015–2024; Second review of progress, 2018*. Delhi PP - Delhi: : World Health Organization. Regional Office for South-East Asia 2018. https://apps.who.int/iris/handle/10665/274310

For peer review only - http://bmjopen.bmj.com/site/about/guidelines.xhtml

4 Van Lerberghe W, Conceição C, Van Damme W, *et al.* When staff is underpaid: Dealing with the individual coping strategies of health personnel. *Bull World Health Organ* 2002;**80**:581–4. doi:10.1590/S0042-96862002000700011

5 Rowe AK, DeSavigny D, Lanata CF, *et al.* How can we achieve and maintain high-quality performance of health workers in low-resource settings ? *Lancet* 2005;**366**. doi:10.1016/S0140-6736(05)67028-6

6 World Health Organization. *The world health report 2006: Working together for health*. Geneva: : World Health Organization 2006.

7 Henderson LN, Tulloch J. Incentives for retaining and motivating health workers in Pacific and Asian countries. *Hum Resour Health* 2008;**6**:18. doi:10.1186/1478-4491-6-18

8 Ahmed SM, Evans TG, Standing H, *et al.* Harnessing pluralism for better health in Bangladesh. *Lancet* 2013;**382**:1746–55. doi:10.1016/S0140-6736(13)62147-9

9 Ahmed SM, Alam BB, Anwar I, *et al. Bangladesh Health System Review*. Dhaka: : World Health Organization 2015.

10 Cometto G, Witter S. Tackling health workforce challenges to universal health coverage: setting targets and measuring progress. *Bull World Health Organ* 2013;**91**:881–5. doi:10.2471/BLT.13.118810

11 Government of Bangladesh. Bangladesh Health Workforce Strategy 2015. Dhaka: 2015.

12 Darkwa EK, Newman MS, Kawkab M, *et al.* A qualitative study of factors influencing retention of doctors and nurses at rural healthcare facilities in Bangladesh. *BMC Health Serv Res* 2015;**15**:1–12. doi:10.1186/s12913-015-1012-z

13 Rawal LB, Joarder T, Islam SMS, *et al.* Developing effective policy strategies to retain health workers in rural Bangladesh: a policy analysis. *Hum Resour Health* 2015;**13**.

For peer review only - http://bmjopen.bmj.com/site/about/guidelines.xhtml

doi:10.1186/s12960-015-0030-6

14 Reynolds JD, Costello T, Edwards MT. The Study of Workload in Child Protective and Child Welfare Services. *Prot Child A Prof Publ Am Hum* 2008;**23**:3–5.https://www.researchgate.net/profile/Robin_Perry3/publication/237430886_A_Critical_Appraisal_of_What_Child_Welfare_Workers_Do_Findings_From_a_Task_Analysis_Study_in_Florida/links/54662f200cf25b85d17f5b5b.pdf#page=4

15 World Health Organization. *Workload Indicators of Staffing Need: User's Manual*. Geneva: : World Health Organization 2010.

16 Lopetegui M, Yen PY, Lai A, *et al.* Time motion studies in healthcare: What are we talking about? *J Biomed Inform* 2014;**49**:292–9. doi:10.1016/j.jbi.2014.02.017

17 Government of Bangladesh. Health Bulletin. Dhaka: 2016.

18 Ritchie J, Lewis J. *Qualitative Research Practice: A Guide for Social Science Students and Researchers*. Thousand Oaks, California: : SAGE 2003.

19 Government of Bangladesh. Bangladesh Essential Health Service Package (ESP). Dhaka: 2016.

20 Ahmed SM, Hossain MA, Chowdhury AMR, *et al.* The health workforce crisis in Bangladesh: shortage, inappropriate skill-mix and inequitable distribution. *Hum Resour Health* 2011;**9**:3. doi:10.1186/1478-4491-9-3

21 Greenglass ER, Burke RJ, Moore KA. Reactions to Increased Workload: Effects on Professional Efficacy of Nurses. *Appl Psychol* 2003;**52**:580–97. doi:10.1111/1464-0597.00152

22 McQuide PA, Kolehmainen-Aitken R-L, Forster N. Applying the workload indicators of staffing need (WISN) method in Namibia: challenges and implications for human

For peer review only

For peer review only - http://bmjopen.bmj.com/site/about/guidelines.xhtml

resources for health policy. *Hum Resour Health* 2013;**11**:64. doi:10.1186/1478-4491-11-64

23 Namaganda G, Oketcho V, Maniple E, *et al.* Making the transition to workload-based staffing: using the Workload Indicators of Staffing Need method in Uganda. *Hum Resour Health* 2015;**13**:89. doi:10.1186/s12960-015-0066-7

24 Burmen B, Owuor N, Mitei P. An assessment of staffing needs at a HIV clinic in a Western Kenya using the WHO workload indicators of staffing need WISN, 2011. *Hum Resour Health* 2017;**15**:9. doi:10.1186/s12960-017-0186-3

25 Ly A, Kouanda S, Ridde V. Nursing and midwife staffing needs in maternity wards in Burkina Faso referral hospitals. *Hum Resour Health* 2014;**12**:S8. doi:10.1186/1478-4491-12-S1-S8

26 Nayebi BA, Mohebbifar R, Azimian J, *et al.* Estimating nursing staff requirement in an emergency department of a general training hospital: Application of Workload Indicators of Staffing Need (WISN). *Int J Healthc Manag* 2017;**0**:1–6. doi:10.1080/20479700.2017.1390182

27 Zaman S. Ladies without lamps: nurses in Bangladesh. *Qual Health Res* 2009;**19**:366–74. doi:10.1177/1049732309331876

28 Ministry of Public Administration. Standard setup by the government for human resources in 10, 20, 31, 50, 100, 150, 200, 250, 500, 500 bed general hospitals at Union, Upazila, District, and Division levels, under Ministry of Health and Family Welfare. 2008.

29 Park CSY. Optimizing staffing, quality, and cost in home healthcare nursing: theory synthesis. *J Adv Nurs* 2017;**73**:1838–47. doi:10.1111/jan.13284

30 Park CSY. Thinking “outside the box”. *J Adv Nurs* 2018;**74**:237–8. doi:10.1111/jan.13312

For peer review only

For peer review only - http://bmjopen.bmj.com/site/about/guidelines.xhtml

31 Park CSY, Park JY. Optimal Safe Staffing Standard for Right Workforce Planning. *J Learn Teach Digit Age* 2019;**4**:42–4. doi:10.1111/jan.13284/full

32 Saville CE, Griffiths P, Ball JE, *et al.* How many nurses do we need? A review and discussion of operational research techniques applied to nurse staffing. *Int J Nurs Stud* 2019;**97**:7–13. doi:10.1016/j.ijnurstu.2019.04.015

Figure Legends

Figure 1: The ways WISN can help in human resource decision-making

Figure 2: Methods applied in each WISN step

Figure 3: Map of Jhenaidah and Moulvibazar Districts with study Upazilas identified

Figure 4: Approach of integration of ESP components in defining workload components of health service activity

For peer review only

For peer review only - http://bmjopen.bmj.com/site/about/guidelines.xhtml

Figure 1

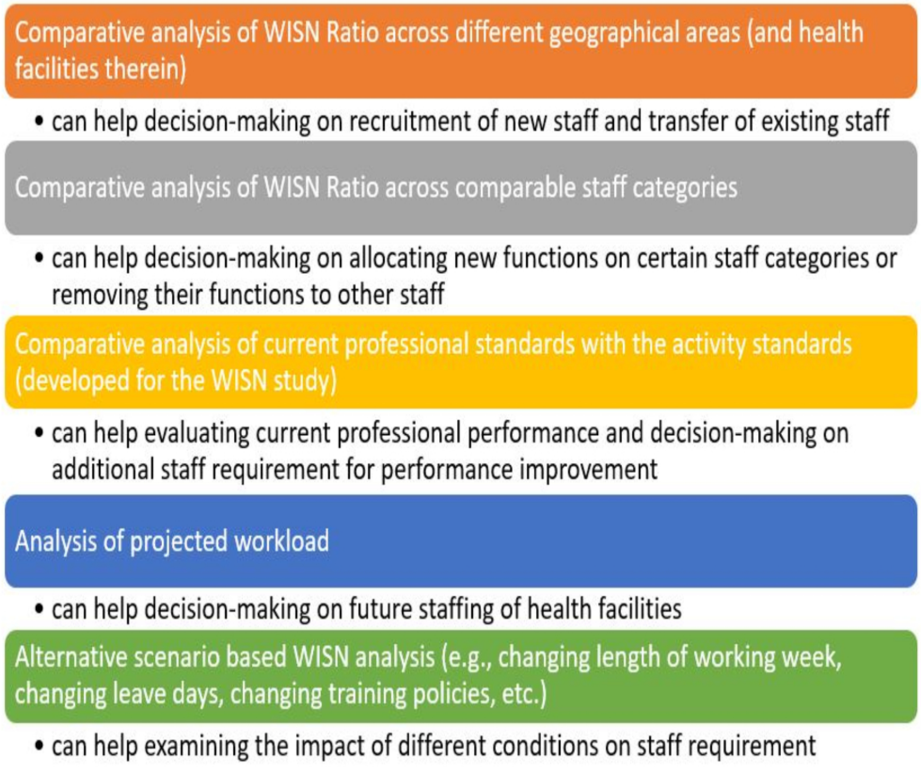

Figure 1. The ways WISN can help in human resource decision-making

The ways WISN can help in human resource decision-making

Figure 2

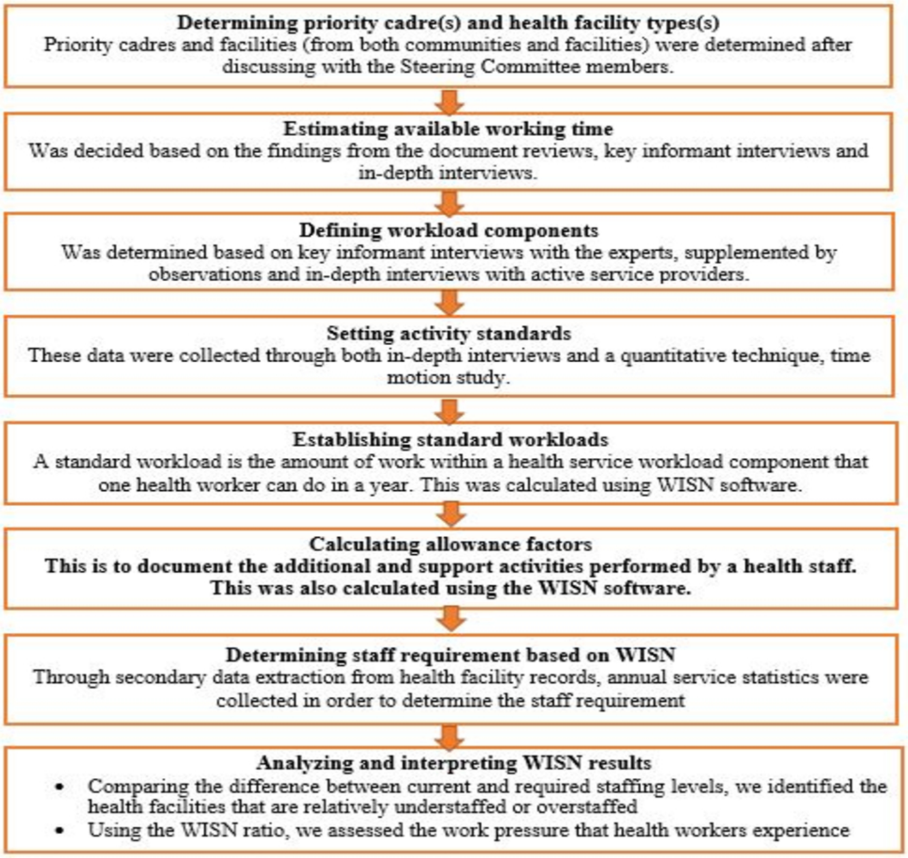

Figure 2 Methods applied in each WISN step

Methods applied in each WISN step

Figure 3

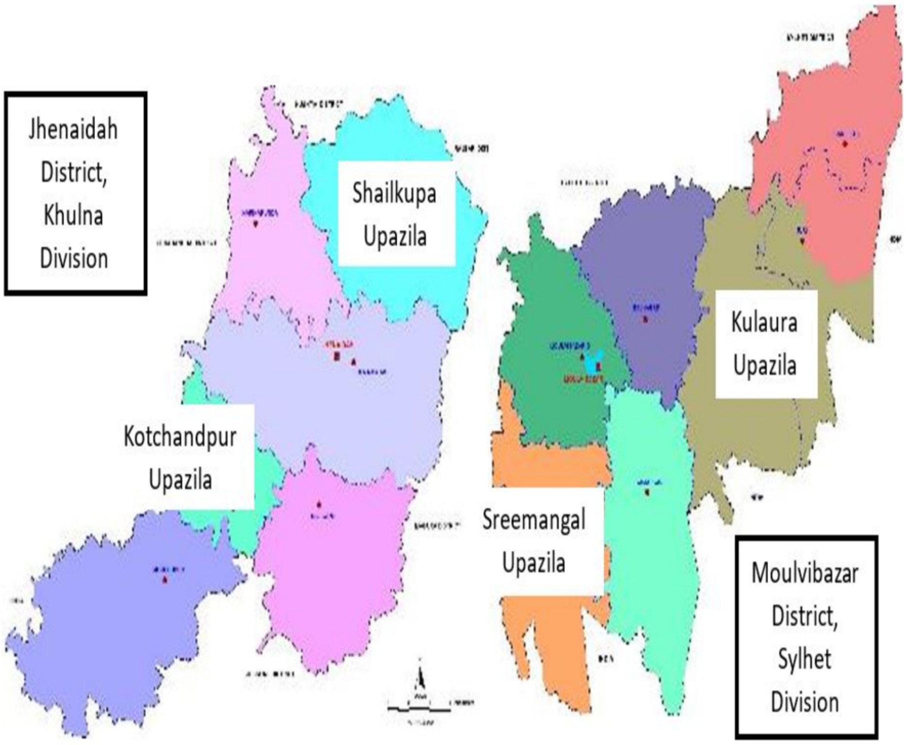

Figure 3 Map of Jhenaidah and Moulvibazar Districts with study Upazilas identified

Map of Jhenaidah and Moulvibazar Districts with study Upazilas identified

Figure 4

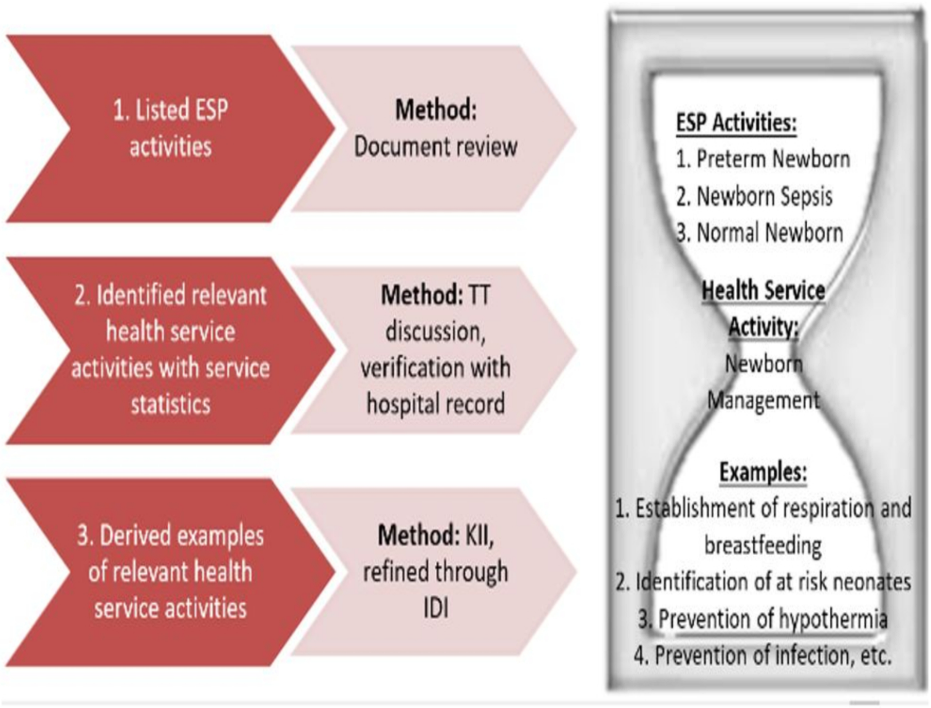

Figure 4 Approach of integration of ESP components in defining workload components of health service activity

Approach of integration of ESP components in defining workload components of health service activity
